# Molecular recognition of RAS/RAF complex at the membrane: Role of RAF cysteine-rich domain

**DOI:** 10.1038/s41598-018-26832-4

**Published:** 2018-05-31

**Authors:** Timothy Travers, Cesar A. López, Que N. Van, Chris Neale, Marco Tonelli, Andrew G. Stephen, S. Gnanakaran

**Affiliations:** 1 Theoretical Biology and Biophysics Group, Los Alamos National Laboratory, Los Alamos, New Mexico 87545 United States; 2 Center for Nonlinear Studies, Los Alamos National Laboratory, Los Alamos, New Mexico 87545 United States; 3 NCI RAS Initiative, Cancer Research Technology Program, Frederick National Laboratory for Cancer Research, Leidos Biomedical Research, Inc., Frederick, Maryland 21702 United States; 4 National Magnetic Resource Facility at Madison, Biochemistry Department, University of Wisconsin-Madison, Madison, Wisconsin 53706 United States

## Abstract

Activation of RAF kinase involves the association of its RAS-binding domain (RBD) and cysteine-rich domain (CRD) with membrane-anchored RAS. However, the overall architecture of the RAS/RBD/CRD ternary complex and the orientations of its constituent domains at the membrane remain unclear. Here, we have combined all-atom and coarse-grained molecular dynamics (MD) simulations with experimental data to construct and validate a model of membrane-anchored CRD, and used this as a basis to explore models of membrane-anchored RAS/RBD/CRD complex. First, simulations of the CRD revealed that it anchors to the membrane via insertion of its two hydrophobic loops, which is consistent with our NMR measurements of CRD bound to nanodiscs. Simulations of the CRD in the context of membrane-anchored RAS/RBD then show how CRD association with either RAS or RBD could play an unexpected role in guiding the membrane orientations of RAS/RBD. This finding has implications for the formation of RAS-RAS dimers, as different membrane orientations of RAS expose distinct putative dimerization interfaces.

## Introduction

The MAPK pathway (RAS/RAF/MEK/ERK) is a cascade of protein-protein interactions that transmits signals from cell-surface receptors to the nucleus, where cellular programs for proliferation and survival are then initiated^1,2^. Mutations of all proteins in the MAPK pathway are observed in human cancer, with high frequencies of KRAS4b mutations in pancreatic, lung, and colorectal cancer^3,4^ and BRAF mutations in melanoma, papillary thyroid, and colorectal cancer^5^. In this context, RAS functions as a binary switch that is converted from inactive RAS-GDP to active RAS-GTP after stimulation of upstream receptors^6,7^. Activated RAS then recruits members of the RAF kinase family^8,9^ to the membrane where they dimerize and become active, thereby propagating the growth signal. RAF proteins bind with high (nanomolar) affinity to RAS via the RAS-binding domain (RBD)^10,11^. There is also experimental evidence that zinc-coordinated cysteine-rich domain (CRD) can bind to RAS independently of RBD in solution^12,13^ with weaker (micromolar) affinity^14^. However, although a crystal structure of the RAS/RBD complex has been solved^15^, no experimentally-derived structure of the RAS/CRD complex is currently available. While significant progress has been made in defining requirements for RAF activation^9^, the precise molecular details underlying the interaction of RAS with RAF via formation of a RAS/RBD/CRD ternary complex at the cell membrane remain unclear. Elucidation of these mechanisms should both improve our understanding of RAF activation in the MAPK pathway and aid in the development of more effective therapies against MAPK-driven cancers.

Experimentally-resolved structures are available for the isolated CRD^16^, RBD^17^, and kinase domain (KD)^18,19^ of RAF. However, structural characterization of full-length RAF is complicated by the presence of two unstructured regions: a linker that connects the CRD and KD (comprising 165–180 residues depending on the RAF isoform^20^), and a disordered N-terminal region that has been implicated in formation of the RAS/RAF complex^21^. The solution structure of the CRD^16^ shows that it adopts a fold similar to that of the C1 domains in protein kinase C (PKC)^22^, with a core β-sheet structure and two relatively long hydrophobic loops that anchor the PKC C1 domains into membranes^23,24^. The CRD also has a preference for inserting into membranes, particularly those containing phosphatidylserine^25^, despite a five-residue deletion in one of its two hydrophobic loops. The CRD could thus fulfill dual roles during RAF activation: anchoring RAF to the membrane while concurrently binding membrane-anchored and activated RAS molecules.

The RBD-independent binding of the CRD to RAS was first shown in solution via *in vitro* ELISA experiments, using the CRD from CRAF (also called RAF1) and non-farnesylated HRAS^12^. This study also showed that the expression of either RBD or CRD in cultured cells blocks the transcription of growth-related genes, presumably by competing with endogenous CRAF for binding to activated RAS. Although C-terminal farnesylation was found to increase the binding of RAS to CRD in solution^14^, the significance of this preference remains unclear at the membrane. Hu and colleagues showed that whereas RBD binding to RAS is GTP-dependent, CRD binding is nucleotide-independent^13^. These investigators also showed that mutation of RAS residues N26 and V45 leads to loss of CRD binding in solution (while not affecting RBD binding), and expression of these RAS mutants impairs CRAF auto-phosphorylation and downstream ERK phosphorylation. This effect is consistent with NMR experiments showing that chemical shift changes occur in RAS residues 23–30 upon RAS/CRD binding^26^. Immunoprecipitation assays on cell extracts have also revealed that CRAF mutations at two CRD residues that coordinate a single zinc ion (C165S and C168S) reduced RAS binding, with cells expressing this double mutant exhibiting decreased KD activation^27^. It was later shown that the membrane affinity of CRAF is not affected by these mutations, as fractionation of sonicated cells showed comparable levels of either wild-type (WT) or mutant CRAF in the pelleted membrane-containing fractions^28^. Similar results have also been obtained using the CRD from the BRAF isoform^29^, thereby indicating that association of the CRD with activated RAS at the cell membrane is relevant for signal transduction in live cells.

In this work, we have used all-atom (AA) and coarse-grained (CG) molecular dynamics (MD) simulations to obtain insights on the structure and dynamics of the RAS/RBD/CRD ternary complex. We focused here on the KRAS4b isoform as this is the most frequently mutated RAS isoform in several human cancers^3,4^, and on the BRAF isoform as it has been found to function as the initial activator of asymmetric RAF heterodimers containing CRAF^19,30–32^ as well as being the most frequent RAF target for mutational activation in human tumors^33,34^. Specifically, we first extensively characterized the membrane binding of the CRD using AA and CG simulations to show that the stabilizing interactions primarily involve embedding of its two hydrophobic loops into the membrane. We then carried out NMR experiments to verify the regions of CRD that associate with the membrane. Next, we used CG MD simulations to probe the membrane interactions and orientations of the RAS/RBD complex. Finally, we investigated how the orientation of the CRD influences the topology of the RAS/RBD/CRD ternary complex and showed that the orientations of RAS and the RBD with respect to the membrane (hereafter membrane orientations) are influenced by which of these two components interacts more with the CRD. Importantly, the various membrane orientations adopted by RAS^35–39^ present different solvent-exposed interfaces on the G domain that have been implicated in the formation of RAS dimers^40,41^, and thus the structure and dynamics of the RAS/RBD/CRD ternary complex presented here can help to improve our understanding of the mechanisms of RAS dimerization and RAS/RAF association that lead to RAF activation.

## Results

### Atomistic model of membrane-anchored RAF-CRD

**Figure 1:**
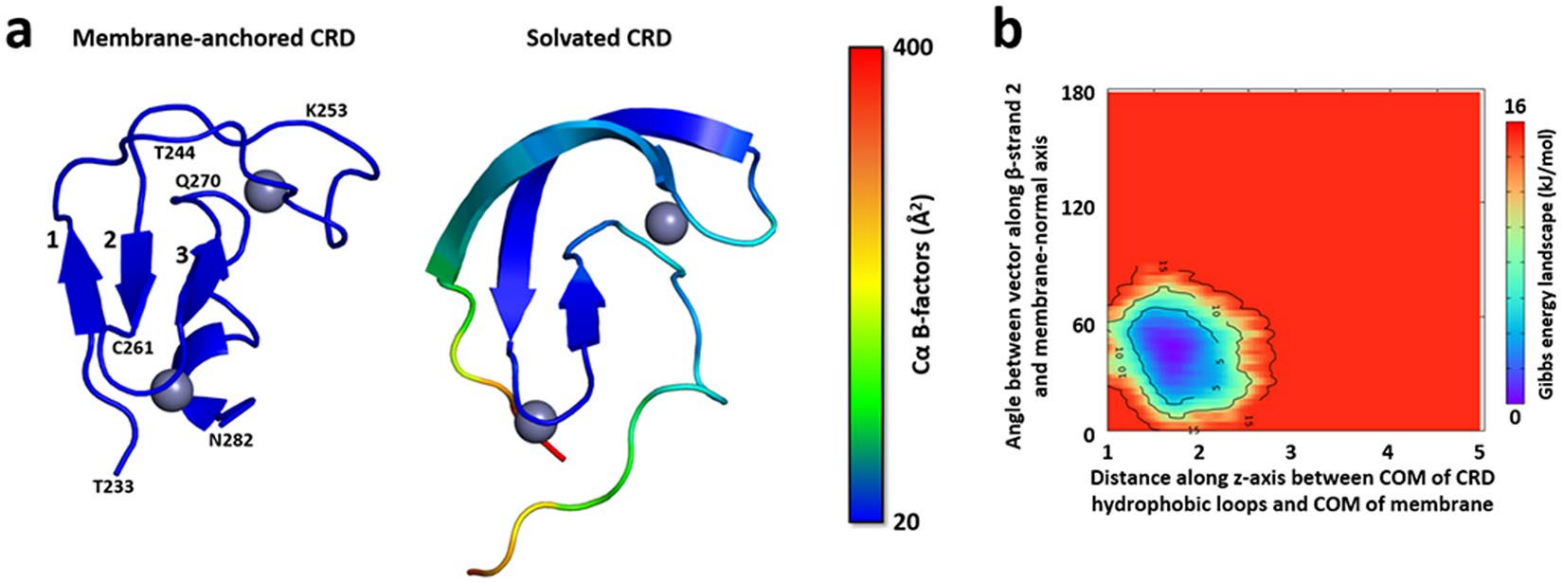
Simulations of membrane-anchored and solvated RAF-CRD. (**a**) Per-residue α-carbon B-factors (Å^2^) from five AA simulations of membrane-anchored CRD (left) and five simulations of solvated CRD (right). Low to high B-factor values are colored from blue to red. Gray spheres denote coordinated zinc ions. The three β-strands comprising the core β-sheet structure are labeled 1–3 in the membrane-anchored structure. N- and C-terminal residues, as well as residues that showed significant chemical shift perturbations in NMR experiments (see Fig. 2a) are also labeled. Typical conformational distortions observed in simulations of solvated CRD, particularly in the two hydrophobic loops (top of the structure), are shown. (**b**) Free energy (kJ/mol) contour map of membrane-anchored configurations of CRD from AA simulations, using as order parameters (i) the distance along the bilayer normal between the COM of both CRD hydrophobic loops and the bilayer COM, and (ii) the angle between a vector defined along β-strand 2 and the bilayer normal (see Supp. Figure 3a).

To assess whether the two hydrophobic loops of the CRD are responsible for its membrane interactions, we performed 1-µs AA simulations of the CRD bound to a lipid bilayer containing 70% POPC (1-palmitoyl-2-oleoyl-*sn*-glycero-3-phosphocholine) and 30% POPS (1-palmitoyl-2-oleoyl-*sn*-glycero-3-phospho-L-serine) after extensive equilibration of membrane lipids around the CRD using a CG Martini representation. Computed B-factors (Fig. 1a left) and root-mean-squared (RMS) fluctuations of atomic positions (Supp. Figure 1a) showed overall fold stability when the CRD was membrane-anchored, including the core β-sheet structure comprised of three β-strands (Supp. Figure 2a). In contrast, simulations of the CRD in solution (i.e., no membrane present) evolved higher B-factors (Fig. 1a right), larger RMS fluctuations (Supp. Figure 1b), and a slight distortion in the domain fold where the sheet structures of β-strands 1 and 2 extended into both of the adjacent hydrophobic loops (Fig. 1a right and Supp. Figure 2b). These results indicate that the membrane anchoring of the CRD by its hydrophobic loops imparts domain stability on the microsecond timescale.

We next looked at how the CRD is oriented when it is anchored to the membrane in these AA simulations. Two order parameters were selected to represent the orientation of the CRD at the membrane (Supp. Figure 3a). The first order parameter, *d*_z_, describes the distance along the bilayer normal between the center of mass (COM) of both hydrophobic loops and the COM of the lipid bilayer, with smaller values indicating deeper membrane penetration of the CRD loops. The second order parameter, *θ*_t_, describes the tilt angle between a vector defined along β-strand 2 and the bilayer normal, with values close to 90° indicating that β-strand 2 is approximately parallel to the membrane surface. Projection of the AA simulation data onto these two coordinates reveals a single free energy basin in which the value of *d*_z_ varied from 1 nm to 2.75 nm and the value of *θ*_t_ varied from 0° to 80° (Fig. 1b). All of these configurations are consistent with a CRD that is anchored to the membrane via its two hydrophobic loops.

To further evaluate whether the membrane binding mode involving CRD loop insertion is the dominant form of CRD at the membrane, we employed a CG approach that provides clear advantages in terms of computational speed. Here, we explored the membrane-CRD interactions formed during unbiased membrane binding of a CRD from aqueous solution. Specifically, the CRD was initially positioned with its COM 5 nm away from the membrane surface (Supp. Figure 3b), and 10 of these simulations were run for 200 µs each. Analysis of these CG runs indicated that the dominant membrane-binding mode indeed involves insertion of both of the CRD hydrophobic loops (Supp. Figure 3c). The first coordinate varied from 1.5 nm to 2.5 nm, while the second coordinate varied from 10° to 100°. The differences in ranges for both coordinates between the CG and AA runs is likely due to the elastic network restraints applied in the CG runs that constrained the backbone conformation of CRD secondary structural elements, while no such restraints were applied to the CRD in the AA runs.

### Experimental verification of RAF-CRD membrane association

**Figure 2:**
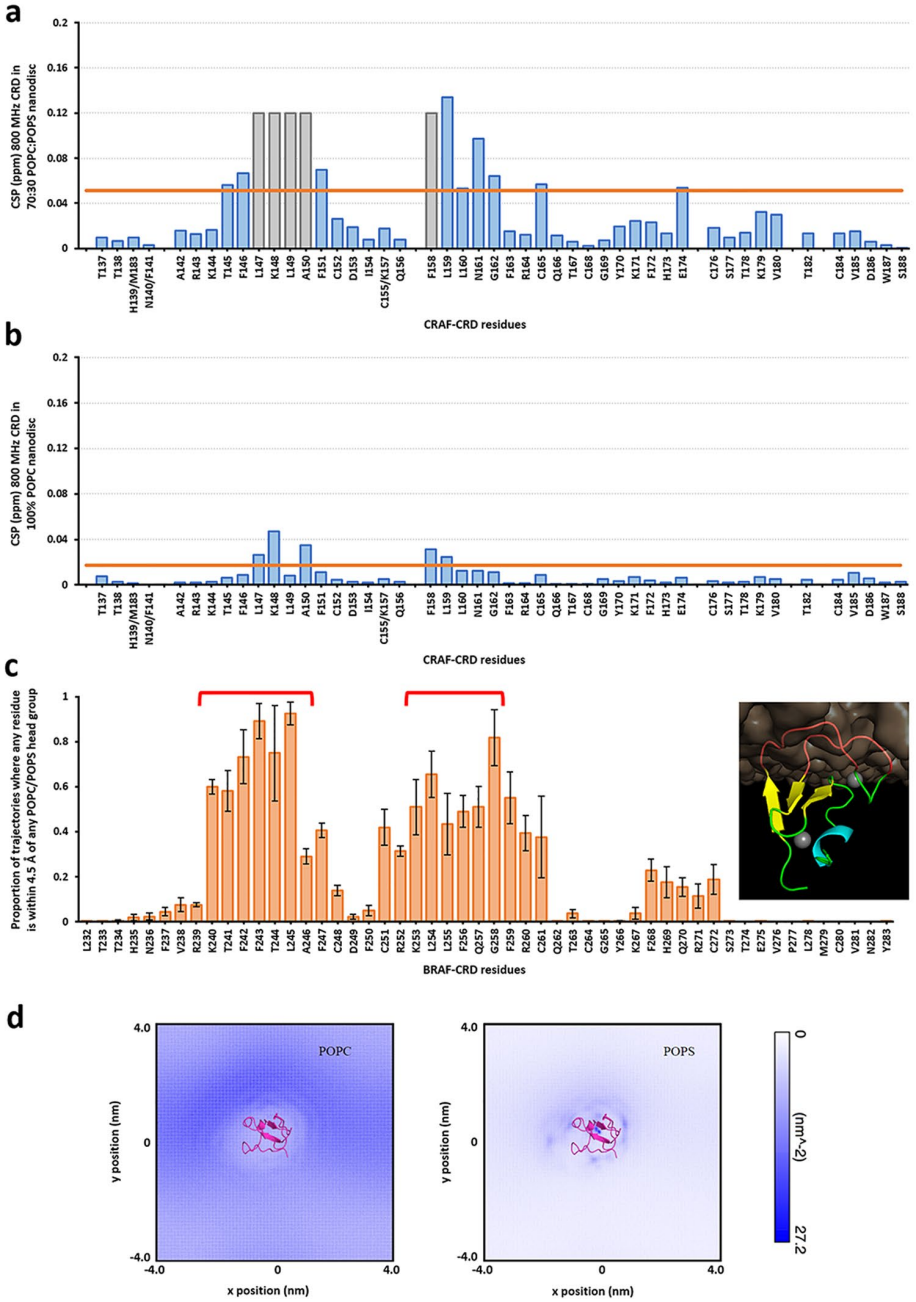
Identification of membrane anchoring residues of RAF-CRD via MD simulations and NMR experiments. (**a**) Per-residue chemical shift perturbation (CSP) profile for CRAF-CRD based on comparison of 800 MHz TROSY spectra for CRD bound to 70%:30% POPC:POPS nanodiscs and for free CRD (see Supp. Figure 4a). Gray bars indicate residues whose peaks broadened beyond detection in the presence of nanodiscs. Orange horizontal line gives average + standard deviation over all measured CSP values in this profile. (**b**) Corresponding per-residue CSP profile for CRAF-CRD based on comparison of 800 MHz TROSY spectra for CRD bound to 100% POPC nanodiscs and for free CRD (see Supp. Figure 4b). Orange horizontal line gives average + standard deviation over all measured CSP values in this profile. (**c**) Per-residue membrane contact profiles for BRAF-CRD from AA simulations, with a higher value indicating a larger proportion of the simulation time in which the residue was making at least one membrane contact. Membrane contacts are defined here as occurring when any heavy atom in a residue is within 4.5 Å of any lipid head group. Red brackets denote the residues belonging to the two hydrophobic loops of CRD. Inset at the right shows a membrane-anchored conformation of CRD via embedding of both its hydrophobic loops (red cartoons). Error bars give the s.e.m. from five simulations. (**d**) Plots of POPC (left) and POPS (right) lipid density (nm^2^), for BRAF-CRD from CG simulations. Only lipids belonging to the leaflet that CRD is anchored to were considered in these calculations. The CRD (magenta cartoons) is shown at the center of both plots in the hydrophobic loop-embedded orientation.

The membrane association of the CRD via its two hydrophobic loops was verified using NMR measurements of CRAF-CRD binding to nanodiscs containing 70%:30% POPC:POPS. Chemical shift perturbations (CSP) of the backbone amides were used to compare the NMR spectra of the CRD alone or in the presence of nanodiscs, and are plotted in Fig. 2. CSPs provide information on the change in the chemical environment of amino acids that result from either direct interaction of the CRD with the membrane or due to CRD conformational changes that result from binding to the membrane. The perturbations were concentrated to two regions that contained positively-charge lysine residues, K148 and K157 (T244 and K253 in BRAF-CRD; see Fig. 1a), flanked by hydrophobic residues. Of note, the peaks for residues L147, K148, L149, A150, and F158 (F243, T244, L245, A246, and L254 in BRAF-CRD) broadened beyond detection in the presence of nanodiscs containing 30% POPS lipids (Fig. 2a and Supp. Figure 4a). This indicates that these residues in the CRD associated tightly to the nanodisc resulting in peak broadening due to the increase in relaxation rate from the apparent increase in molecular weight. In addition, C165 and E174 (C261 and Q270 in BRAF-CRD) also showed significant perturbations when the CRD was bound to nanodiscs. We also performed measurements for the CRD bound to 100% POPC nanodiscs, and while CSPs were still observed for a number of peaks, their magnitude was significantly decreased compared with the CSPs observed for the POPS-containing nanodiscs (Fig. 2b and Supp. Figure 4b). This indicates that the CRD has a higher binding affinity for POPS-containing nanodiscs.

We next analyzed both AA and CG simulations of a membrane-anchored CRD for properties that can track the NMR measurements in more detail, particularly the interactions between the CRD and the membrane in the loop-embedded orientation. One such property is the fraction of simulation time that each residue is in contact with any lipid, which was computed here using a cutoff distance of 0.45 nm between heavy atoms of each residue with any lipid head group. The AA (Fig. 2c) and CG (Supp. Figure 5) simulations showed that residues from both hydrophobic loops are primarily involved in making lipid contacts, consistent with the NMR measurements. We note that the AA and CG simulation membrane contact profiles do not exactly match the CSP profile using POPS-containing nanodiscs (Fig. 2a), as the former were computed only from simulations of membrane-anchored CRD while the latter is based on differences between solution and membrane-anchored CRD.

The AA and CG simulations were consistent in showing that on average around 6–8 POPS lipids can be found near the CRD (Supp. Fig. 6a). To check if this means that the CRD preferentially interacts with POPS lipids, we computed from the more extensive CG runs a 2D density map of POPS and POPC lipids in the leaflet to which the CRD anchors (Fig. 2d). This clearly shows the clustering of POPS lipids around the CRD, while POPC lipids were more evenly distributed throughout this leaflet. We also computed a time profile for the proportion of either lipid around the CRD, using the same cutoff distance of 0.45 nm as earlier. The protein-distal leaflet (i.e., the leaflet that does not have CRD embedded) shows that the average POPC:POPS proportion stays at 70%:30% (with a standard deviation of around ±10% for both values) (Supp. Fig. 6b top). The protein-proximal leaflet (i.e., the leaflet with embedded CRD), on the other hand, shows an increased amount of POPS lipids around the CRD with an average POPC:POPS proportion of 60%:40% (standard deviation also around ±10%) (Supp. Fig. 6b bottom).

### KRAS4b G domain orientational landscape at the membrane with RAF-RBD bound

We next looked at the membrane dynamics of the tightly-bound RAS/RBD complex on a lipid bilayer containing 70%:30% POPC/POPS. For this evaluation, we performed only CG simulations as the goal was to obtain efficient sampling of membrane orientations. The KRAS4b/BRAF-RBD complex was built via homology modeling to the crystal structure of the HRAS/CRAF-RBD complex^15^, and the entire complex was initially positioned such that the COM of the RAS G domain and RBD were 4 nm and 7 nm, respectively, away from the membrane except for the anchoring farnesyl at the C-terminus of the RAS hypervariable region (HVR). To characterize the membrane orientations, we followed a similar procedure as in the case of the CRD simulations, however using a different set of order parameters to define orientations. In particular, we measured the tilt of the G domain away from the bilayer normal, *θ*_t_, vs. the azimuthal angle at which that tilt occurs (*i.e*., the rotation), *θ*_r_. Definitions of *θ*_t_ and *θ*_r_ are provided in the Methods and illustrated in Supp. Fig. 7.

**Figure 3:**
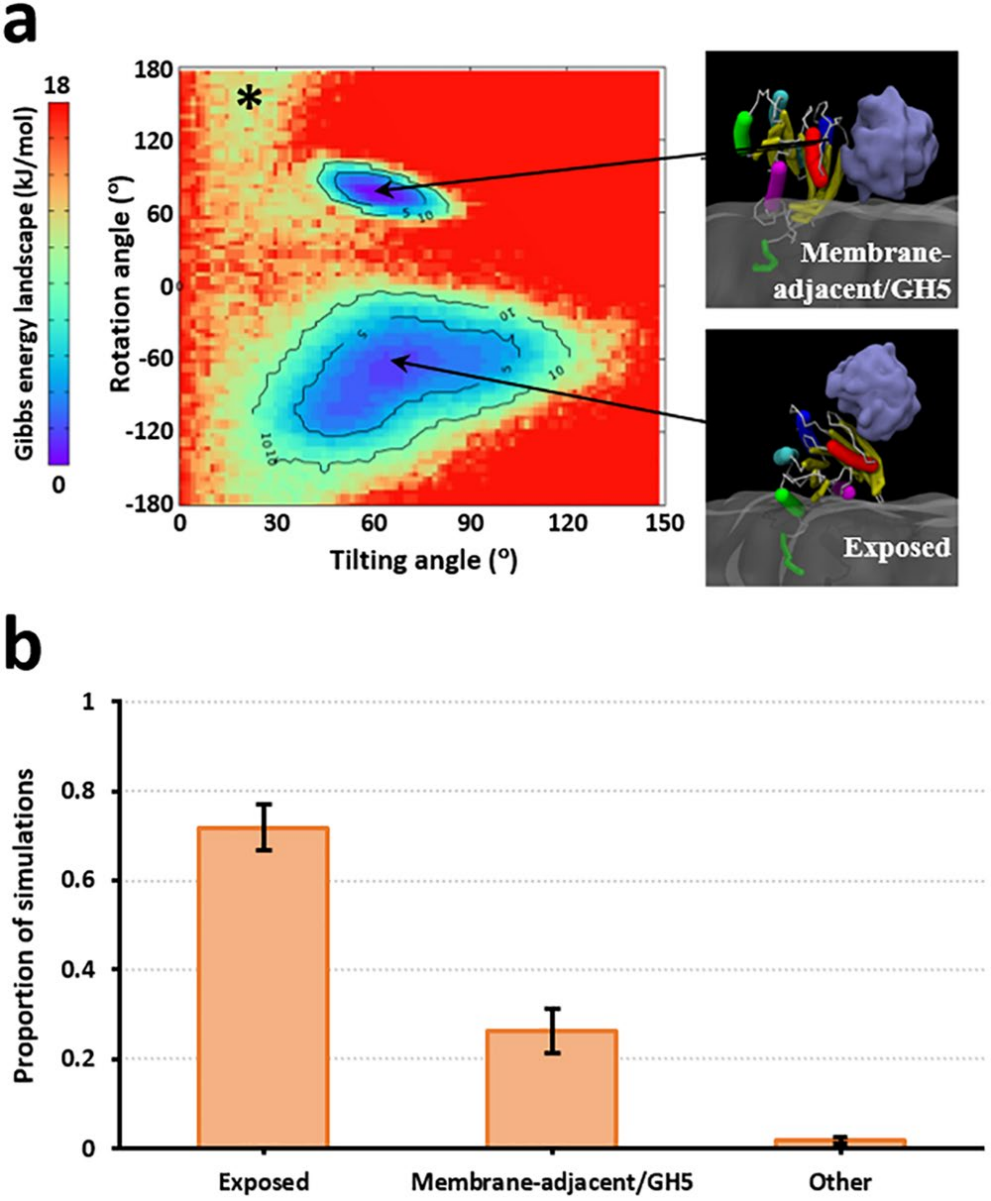
RAS/RBD adopts two dominant orientations at the membrane. (**a**) Free energy (kJ/mol) contour map of membrane-anchored configurations of RAS/RBD from CG simulations, using as order parameters (i) the tilt of the G domain away from the bilayer normal, and (ii) the azimuthal angle at which this tilt occurs (see Supp. Fig. 7). Insets at the right show sample snapshots belonging to each of the two dominant membrane orientations observed in the simulations. RAS is shown using a multicolored tube representation, while a surface depiction is used for the RBD. The asterisk indicates a minor basin that appears to connect the two larger basins. (**b**) Bar plot showing the proportion of simulation snapshots belonging to the membrane orientations observed in the CG simulations of membrane-anchored RAS/RBD. Error bars give the s.e.m. from ten simulations.

After projecting these two correlated angles, we obtained a free energy contour map (Fig. 3a; see Supp. Fig. 8 for a population-based contour map) that clearly shows two dominant basins: the first basin is centered at around 60° tilt and 100° rotation angles, while the second basin is centered at around 75° tilt and −60° rotation angles. The latter basin comprised around 72% of configurations sampled in the CG simulations (Fig. 3b), and features an orientation where helices 4/5 of the G domain are in close proximity to the water-lipid interface of the bilayer, and with the RBD-binding site exposed to solvent (hereafter the “Exposed” orientation). The other basin comprised around 26% of configurations sampled, has helix 5 and the β-strands of the G domain near the membrane, and has the RBD-binding site still solvent-exposed but now adjacent to the membrane (hereafter the “Membrane-adjacent/GH5” orientation, to denote that helix 5 of the G domain is also near the membrane). The insets in Fig. 3a show sample structures belonging to both of these dominant orientations. The remaining 2% of configurations sampled includes a minor basin (marked by an asterisk in Fig. 3a) that appears to connect the two larger basins. When RAS is in the Exposed orientation, no membrane contacts are made by the RBD (Supp. Fig. 9a); however, when RAS is in the Membrane-adjacent/GH5 orientation, the RBD makes membrane contacts and shows a preference for binding POPS lipids (Supp. Fig. 9b). A more detailed analysis of the RAS/RBD snapshots in the Membrane-adjacent/GH5 orientations (Supp. Fig. 10) showed that most of the RBD residues making contacts with the membrane come from the interface of the RBD β-sheet that is opposite to RBD helix 1.

### Ternary complex models of RAS/RBD/CRD based on solution data of RAS/CRD association

**Figure 4:**
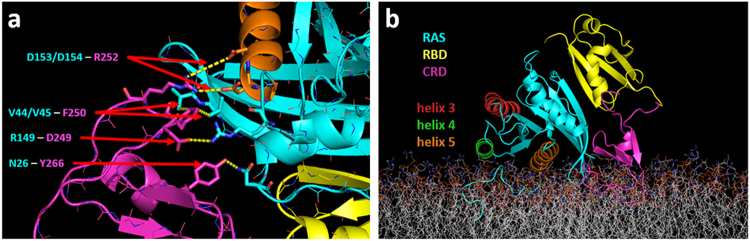
RAS/RBD/CRD ternary complex model based on RAS/CRD association observed in solution studies. (**a**) Snapshot from the AA simulation of the solvated ternary complex model S1 (see Supp. Fig. 11), focusing on the binding interface between RAS and CRD. The CRD and RBD are shown in pink and yellow cartoons, respectively. RAS is shown in cyan cartoons, but with helix 5 colored orange. Stable interactions between RAS and CRD that were observed in the 1-µs simulation are highlighted, and include RAS residues N26 and V45 (see contact maps in Supp. Fig. 12a). (**b**) Snapshot from one of the AA simulations of membrane-anchored ternary complex model S1, showing RAS adopting the Exposed orientation. The CRD, RBD and RAS are colored as in (**a**), but with helices 3, 4, and 5 of the RAS colored red, green, and orange, respectively, in order to show their disposition relative to the membrane surface.

To probe how the CRD influences the membrane orientations of RAS/RBD, we used a docking-based approach to construct candidate models of the RAS/RBD/CRD ternary complex at the membrane. These models were then filtered based on three experimentally-derived criteria: (i) the two hydrophobic loops of CRD must be accessible to allow for membrane anchoring; (ii) the C-terminus of the RBD must be sufficiently close to the N-terminus of the CRD that these domains can be connected by their intervening four-residue linker; and (iii) the CRD must contact the surface of RAS that presents residues N26 and V45, which have been shown experimentally to be important for CRD binding^13^. After applying these filters, only three models remained for the RAS/RBD/CRD ternary complex (labeled as models S1, S2, and S3 in Supp. Fig. 11), all of which can be anchored to the membrane with CRD adopting an orientation that has both hydrophobic loops embedded in the membrane and such that the RAS G domain adopted the Exposed orientation with the RBD not interacting directly with the membrane. We ran 1-µs AA simulations for each of the three docked models in solution (i.e., no membrane present) to assess the stability of each model and to verify that stable contacts are made between the CRD and residues N26 and V45 of RAS. The simulations for model S1 formed a stable contact between the side chains of RAS N26 and CRD Y266, as well as hydrophobic contacts between RAS V44/V45 and CRD F250 (Fig. 4a). In addition, stable salt bridges were also detected between RAS R149 and CRD D249, and between RAS D153/D154 and CRD R252. In contrast, intermittent interactions with only RAS N26 were observed for simulations of model S2, while CRD detached from the complex in simulations of model S3. Based on these observations, only model S1 was used to build models of the membrane-associated ternary complex.

We anchored the S1 model of the ternary complex onto a membrane bilayer containing 70%:30% POPC:POPS via embedding of the two hydrophobic loops of CRD, which dictated the Exposed orientation for the RAS/RBD complex (Fig. 4b). The membrane-bound model in this orientation was then assessed by five independent 1-µs AA simulations. As described in the Methods, extensive equilibration of lipids around the ternary complex was accomplished via prior CG simulations. An analysis of contacts between RAS and CRD in these AA runs showed that the intermolecular contacts observed in the solution-only simulations (Fig. 4a**)** were maintained in three of the membrane-bound simulations (Supp. Fig. 12a). For the other two runs, one showed the CRD moving towards RAS N26 and breaking the RAS V45 contact, while the other conversely showed the CRD moving towards RAS V45 and breaking the RAS N26 contact (Supp. Fig. 13). Because of the limited sampling of protein complex conformations in these AA simulations, we cannot conclude from these runs that the RAS/CRD interactions inferred from measurements done in solution are stable in the context of a membrane. We also looked if CRD made any contacts with RBD, and found that except for some transient interactions involving the N- and C-termini of the CRD, there were no persistent contacts formed between the CRD and the RBD in these simulations (Supp. Fig. 12b). Mapping of the tilt and rotation angles of the RAS G domain relative to the membrane shows that the Exposed orientation is maintained in these 1-µs simulations (Supp. Fig. 14a). In terms of the distribution of POPS lipids around each component of the RAS/RBD/CRD ternary complex (Supp. Fig. 15), around 7 POPS lipids clustered around the CRD, similar to the behavior in simulations of membrane-bound CRD alone (Supp. Fig. 6a). The highest distribution of clustered POPS lipids was found around the RAS HVR, which has a polybasic sequence in the KRAS4b isoform.

To ensure that these observations are not biased by the initial membrane anchoring of the complex in these AA runs, we ran CG simulations of the docked model such that the COM of the RAS G domain, RBD, and CRD were initially positioned 5 nm, 6.5 nm, and 8 nm away from the membrane, respectively, except for the anchoring farnesyl at the C-terminus of the RAS HVR. This also allowed us to check if other membrane orientations are possible given this particular topology of the ternary complex. Elastic network restraints were included during these CG simulations to maintain the conformation of each domain as well as the overall topology of the ternary complex. Measurements of the distance between the COM of the CRD and the COM of the bilayer showed that after an initial first passage time that ranged from 5 to 30 µs, the CRD embedded into the membrane in all ten CG trajectories (Supp. Fig. 16a), via membrane insertion of its two hydrophobic loops. These CG simulations also showed that once the CRD became embedded in the membrane, it attracted POPS lipids (Supp. Fig. 16b), and the ternary complex remained in the Exposed orientation with embedded CRD loops and no direct RBD-membrane interactions for the remainder of the 100 µs simulation time (Supp. Fig. 14b). Although the restraints between CRD and RAS imposed by the elastic network may have prevented the RAS G domain from adopting additional orientations at the membrane surface, these were necessary to make our approach computationally tractable.

### Ternary complex models of RAS/RBD/CRD with no pre-defined RAS/CRD association

Our initial approach for modeling the RAS/RBD/CRD ternary complex relied on selecting only those models where the CRD associated with RAS as suggested by solution studies. We next explored ternary complex models where the mode of association between RAS and CRD was not imposed as an initial condition. In these simulations, the CRD was positioned away from RAS/RBD by 2 nm while anchored to the membrane and still attached to the RBD via the native 4-residue linker (Supp. Fig. 17). Five independent 1-µs AA simulations were then performed, to evaluate if and how the CRD would approach RAS/RBD to form a ternary complex. Two of these simulations showed the CRD approaching RAS/RBD to form a ternary complex that resembled the docked model and had similar CRD contact profiles with both RAS and the RBD (see Fig. 4a and Supp. Fig. 12). In the third simulation, the CRD approached RAS near N26 of its G domain without making interactions to the interface near V45 (see left-hand side of Supp. Fig. S13), while another simulation showed the opposite and had CRD approaching the interface near V45 without making interactions near N26 (see right-hand side of Supp. Fig. S13). The final run did not show CRD approaching RAS/RBD on the simulation timescale.

**Figure 5:**
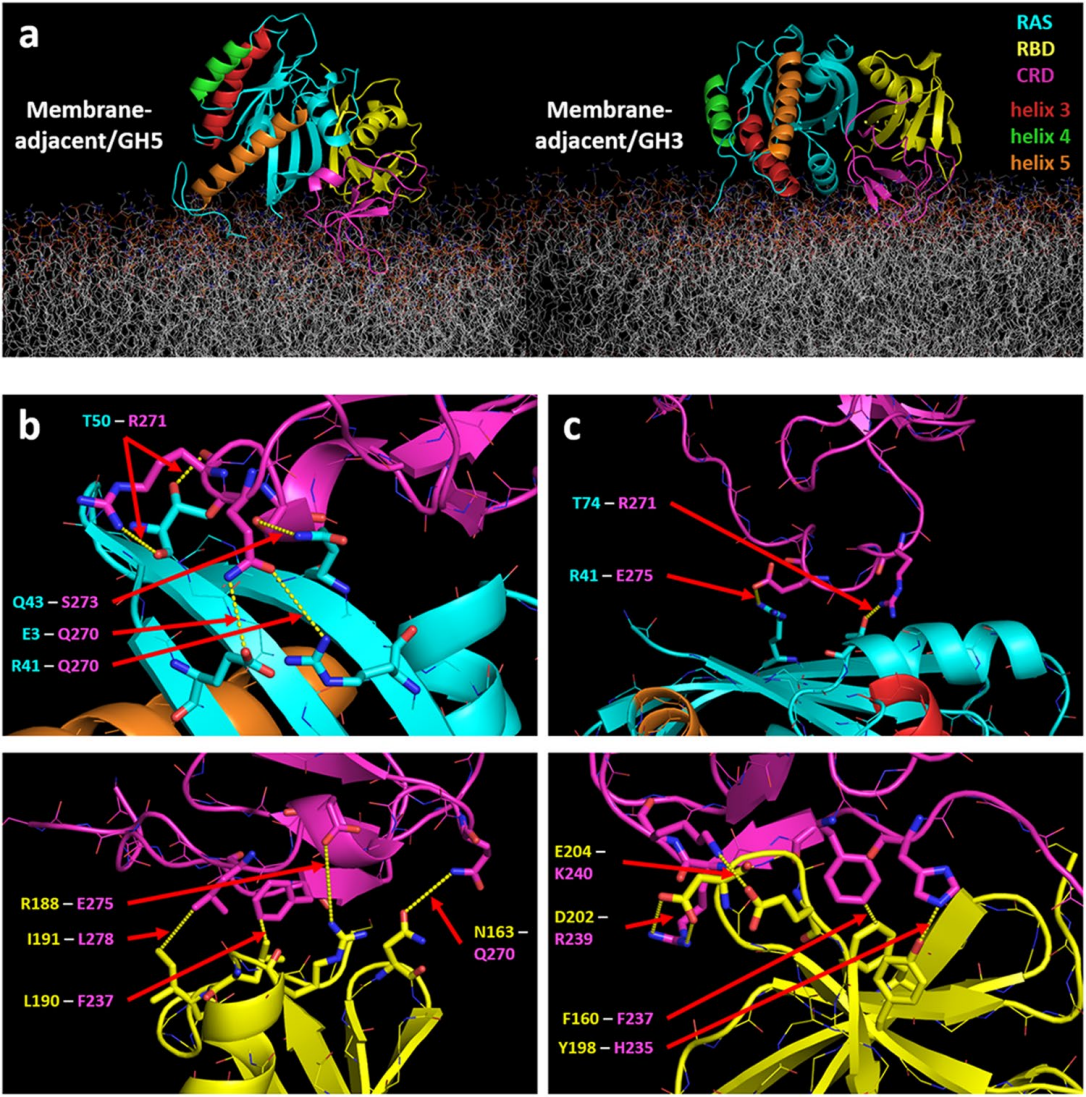
RAS/RBD/CRD ternary complex models based on CG simulations with no pre-defined RAS/CRD association. (**a**) Snapshots showing the Membrane-adjacent/GH5 (left) and membrane-adjacent/GH3 (right) orientations observed in the CG simulations. The CRD, RBD and RAS are colored as in Fig. 4, but with helices 3, 4, and 5 of the RAS colored red, green, and orange, respectively, in order to show their disposition relative to the membrane surface. (**b**) Residue contacts between RAS/CRD (top) and between RBD/CRD (bottom) for the Membrane-adjacent/GH5 orientation are highlighted (see contact maps in Supp. Fig. 18a). (**c**) Residue contacts between RAS/CRD (top) and between RBD/CRD (bottom) for the Membrane-adjacent/GH3 orientation are highlighted (see contact maps in Supp. Fig. 18b).

The varying CRD association results seen in different AA runs may be due to a weak specificity of CRD for RAS, exacerbated by the limited sampling of protein conformations attained in these simulations. To extend the timescales at which we probed the domain interactions and orientations after unbiased binding between the CRD and the RAS/RBD complex, we repeated these simulations using a more efficient CG approach. These CG simulations showed two other orientations being sampled that pulled the RBD closer to the membrane. One of these orientations corresponds to the Membrane-adjacent/GH5 orientation observed earlier in the CG RAS/RBD simulations, where helix 5 of the G domain is near the membrane (Fig. 5a left). The other orientation is also membrane adjacent, but instead has helix 3 of the G domain near the membrane (hereafter the “Membrane-adjacent/GH3” orientation) (Fig. 5a right). The latter orientation in fact corresponds to the minor basin that connected the Exposed and Membrane-adjacent/GH5 orientations in the CG RAS/RBD simulations (basin marked by an asterisk in Fig. 3a). For the current CG simulations, however, the presence of the CRD caused the Membrane-adjacent/GH3 orientation to be sampled more frequently. An analysis of protein domain contacts showed the CRD making more contacts with the RAS G domain in the Membrane-adjacent/GH5 orientation compared to Membrane-adjacent/GH3 orientation (Fig. 5b,c top, Supp. Fig. 18a and b top), which were fewer and distinct from the RAS/CRD contacts seen in the docked model (Fig. 4a, Supp. Fig. 12a). In contrast, the CRD made more contacts with the RBD in both Membrane-adjacent orientations (Fig. 5b,c bottom, Supp. Fig. 18a and b bottom) compared to the docked model (Supp. Fig. 12b). These RAS/CRD and RBD/CRD contacts observed in the CG runs were also observed in AA simulations using backmapped configurations from both Membrane-adjacent orientations (Supp. Fig. 19). Compared to the Membrane-adjacent/GH5 orientation from the CG RAS/RBD runs (Supp. Fig. 9b), the RBD in these RAS/RBD/CRD simulations was not pulled as close to the membrane as evidenced by the lower number of lipid contacts observed (Supp. Fig. 20). Around 7 POPS lipids were found to cluster around CRD in the Membrane-adjacent/GH5 orientation, while slightly more (around 8 POPS lipids) clustered around CRD in the Membrane-adjacent/GH3 orientation (Supp. Fig. 15). Interestingly, compared to the docking-based ternary complex model, both Membrane-adjacent orientations attracted fewer POPS lipids around the RAS G domain (Supp. Fig. 15).

## Discussion

We have extensively characterized the CRD, in solution or anchored to a 70%:30% POPC:POPS membrane, using AA and CG MD simulations (Fig. 1). These simulations showed that membrane anchoring of CRD occurs through the embedding of its two hydrophobic loops into the membrane. This binding mode of CRD to the membrane was confirmed by NMR experiments with CSPs mainly occurring for those residues belonging to either of the CRD hydrophobic loops (Fig. 2). Additionally, these experiments showed that the association of the CRD to membranes is increased in the presence of POPS lipids, and our simulations in turn indicated that these anionic lipids have a propensity to cluster around the CRD. This clustering has also been observed in simulations of the homologous C1 domains of PKC using anionic DOPS lipids^42^. The CRD contains seven basic arginine and lysine residues that form a “belt” around the two hydrophobic loops (Supp. Fig. 21), and interactions of these residues with the lipid head groups can account for the attraction of anionic lipids. We are aware that our simulations were performed using BRAF-CRD while the NMR measurements were done for CRAF-CRD. The high sequence similarity between both CRD isoforms (74.5% identity, 97.9% similarity) (Supp. Fig. 22a) suggests that they may adopt the same fold as well as the same membrane-binding mode. Although it remains unclear whether sequence variation among CRD isoforms functionally constitutes minor tweaking, major changes, or irrelevant evolutionary drift, we note that the two C1 domains of PKC have even more sequence divergence with RAF-CRD than exists among RAF isoforms and yet the C1 domains have also been shown via experiments and simulations to adopt the same fold and membrane binding mode^22,42^ as we have presented here for BRAF-CRD and CRAF-CRD.

We then wanted to explore how CRD-membrane association could impact the RAS/RBD/CRD ternary complex. To do this, we first evaluated the membrane orientations of the RAS/RBD binary complex. In our CG simulations, the entire RAS/RBD complex was effectively considered as a single tightly bound unit, which is a reasonable assumption since the high nanomolar binding affinity between RAS and RBD has been well documented^10,11^. Additionally, the RAS-binding interface of the RBD is evolutionarily conserved between BRAF and CRAF isoforms (Supp. Fig. 22b). Our simulations showed that the RAS/RBD heterodimer tumbles between two major orientations (Fig. 3). The Exposed orientation has the RBD away from the membrane while the Membrane-adjacent/GH5 orientation has the RBD making membrane interactions. Both orientations have been previously identified in an NMR study of KRAS4b interacting with nanodiscs containing 70%:30% POPC:POPS^39^. The dominant orientation was also found to switch between both orientations, depending on the particular isoform of bound RBD^39^: (i) ARAF-RBD has a net positive charge on its membrane binding interface and shifts RAS/RBD more to the Membrane-adjacent/GH5 orientation, while (ii) RalGDS-RBD has a net neutral charge on the corresponding interface and shifts RAS/RBD more to the Exposed orientation. The corresponding interface on BRAF-RBD has a net neutral charge (Supp. Fig. 23) and consistent with the observations for RAS/RalGDS-RBD complex we found here that the Exposed orientation was sampled more in our CG simulations of the RAS/BRAF-RBD complex. CRAF-RBD has a net positive charge on this interface (Supp. Fig. 23), and we therefore predict that this will shift RAS/RBD toward to the Membrane-adjacent/GH5 orientation.

With details about the membrane orientations of RAS/RBD binary complex in hand, we proceeded with modeling the RAS/RBD/CRD ternary complex. The goal here was to explore the potential conformations of the membrane-anchored ternary complex provided that (i) CRD is anchored to the membrane via its two hydrophobic loops, and (ii) RAS/RBD is free to sample either of the membrane-anchored orientations observed earlier. These considerations are carried out with one critical question – does the CRD bind to RAS in such a complex or not? Our first model relied on solution experiments^13^ that suggested two residues from RAS, N26 and V45, potentially interacting with the CRD in the ternary complex. In the corresponding membrane-anchored model and simulations, we ensured that these two RAS residues are indeed making interactions with the CRD. This model, based on atomistic docking of the CRD to the RAS/RBD crystal structure, shows the RAS G domain in an Exposed orientation with the RBD away from the membrane (Fig. 4). AA simulations of this model show CRD residues Y266 and F250 interacting with RAS residues N26 and V45, respectively. This ternary complex model thus provides a testable prediction to see if mutations of CRD residues Y266 and F250 can inhibit RAS/CRD binding. In addition, several salt bridge interactions (RAS R149 – CRD D249 and RAS D153/D154 – CRD R252) can also be explored experimentally for their impact on RAS/CRD binding.

Alternatively, we considered models where no such direct interactions between RAS and CRD were imposed. Both AA and CG simulations were started with membrane-anchored CRD initially positioned away from RAS/RBD. The goal here was to explore whether those interactions mentioned above are specific enough to drive the CRD to reconnect with RAS. However, we did not observe such a strong specificity between RAS and the CRD in these simulations. The AA simulations, which are limited by short time scales, did show tendencies for CRD to become proximal to RAS, however these associations were weak and non-specific. Restricted by the short linker between RBD and CRD and the stronger binding between RAS and RBD, any weak interactions between RAS and CRD could just be incidental in the context of a nearby membrane. With CG simulations that allow for more extensive sampling of the ternary complex over longer time scales, we found two dominant interconverting models of the membrane-anchored ternary complex (Fig. 5) that had the RAS G domain adopting related Membrane-adjacent orientations such that the RBD was close enough to the bilayer to make direct membrane interactions. Interestingly, we found that in contrast to the docked model that showed CRD interacting mainly with RAS and forming only transient interactions with RBD (Supp. Fig. 12), both of these Membrane-adjacent models made more persistent interactions between the CRD and different regions of the RBD (Supp. Fig. 18). These results suggest that the topology and membrane orientations of the ternary complex are altered depending on whether CRD is primarily interacting with either the RAS G domain or the RAF-RBD.

In summary, we have provided atomistic details on the membrane anchoring of RAF-CRD using a combination of simulations and experiments. Both approaches indicate that anionic lipids facilitate the membrane anchoring of the CRD. Encouraged by these findings, we then explored the effect that membrane anchoring of CRD could have on the RAS/RBD/CRD ternary complex. Our results suggest that the manner in which CRD associates with the membrane affects the topology and the membrane orientations of its components, as well as the exposure of putative RAS dimerization interfaces^40,41^. When the CRD is primarily interacting with RAS, the RAS G domain adopts the Exposed orientation in which the putative dimerization interface between helices 3/4 is accessible. In contrast, when the CRD is primarily interacting with the RBD, this causes a shift towards two related Membrane-adjacent orientations of the RAS G domain that both expose putative interfaces between helices 3/4 and helices 4/5 although to different extents. We note that changes in the membrane lateral pressure due to RAS/RAF adsorption are not expected to have any dramatic effects on other physical properties (such as curvature/bending) of the membrane patches used in our simulations. This is because of the small size and simple composition of our simulated patches, as well as the fact that our simulations do not consider protein aggregation/crowding. We also note that it was recently shown, using fluorescence correlation spectroscopy and single-molecule tracking, that KRAS4b appears to lack intrinsic dimerization capability on supported lipid bilayer membranes^43^. The RAS/RBD/CRD models should therefore be useful in elucidating how RAS dimerization and RAS/RAF binding can occur concurrently and lead to RAF activation.

## Methods

### System setup

#### All-atom (AA) models of RAF-CRD

Structural models of BRAF-CRD were based on the first conformer from the solution NMR structure of the CRAF-CRD (PDB 1FAQ)^16^ with *in silico* mutagenesis using PyMOL^44^ to match the sequence of BRAF-CRD. Each of the two zinc ions bound to the CRD are coordinated by three anionic cysteines (thiolates) and one histidine (protonated at the epsilon nitrogen). The CHARMM36 force field^45,46^ contains parameters for thiolate^47^ as well as harmonic bond and angle potentials to maintain the coordination geometry around zinc. For the system containing an isolated CRD in aqueous solution (hereafter the solution CRD system), TIP3P water molecules^48^ were added to fill a rhombic dodecahedral box around the CRD, with a minimum distance of 1.2 nm from any protein atom to any edge of the simulation unit cell. For the membrane-anchored CRD system, the two hydrophobic loops of the CRD were embedded into a lipid bilayer containing 64 lipids per leaflet with a composition of 70%:30% POPC:POPS that was built using the Membrane Builder module of CHARMM-GUI^49,50^. Monovalent K^+^ and Cl^−^ ions were added to both systems in order to neutralize the system charge and to reach a physiological ionic strength of 150 mM.

#### Coarse-grained (CG) model of RAF-CRD for spontaneous membrane association

The AA coordinates of the BRAF-CRD model were transformed into CG beads using parameters based on the MARTINI 2.2 force field^51^. An elastic network was applied to the entire protein backbone in order to preserve the internal secondary structure^52^. A CG membrane containing 70%:30% POPC:POPS was constructed using the *insane* script^53^. To be more consistent with physical properties computed from atomistic CHARMM36 runs, the CG Martini parameters were reparametrized as discussed in the **Supporting Methods**. The CG structure of the CRD was initially placed with its center of mass (COM) 5 nm away from the surface of the bilayer. The entire system was solvated using a total of 5000 CG water beads, and Na^+^ and Cl^−^ ions were added to neutralize the excess charges and mimic the physiological ionic strength of 150 mM.

#### CG models of RAS/RBD for spontaneous membrane association

To study the orientations of RAS/RBD at the membrane, we prepared a system using the MARTINI 2.2 force field^51^. The crystal structure of the complex between HRAS/CRAF-RBD (PDB 4G0N)^15^ was used to build a homology model of KRAS4b/BRAF-RBD via *in silico* mutations using PyMOL^44^, followed by transformation of the atomic coordinates to CG beads. In order to avoid large structural deviations of the RAS/RBD complex, an elastic network^52^ connecting the CG beads within RAS, within RBD, and between RAS/RBD were incorporated (excluding residues 171–185 of the RAS HVR). In order to accurately represent the dynamics of RAS, the CG representation of the farnesyl group was parametrized based on AA simulations as discussed in the Supporting Methods. These AA simulations were conducted with standard CHARMM36 parameters, except for the use of new AA parameters for the farnesyl moiety (Chris Neale, personal communication). The farnesyl group of RAS was embedded in a lipid bilayer containing 70% POPC and 30% POPS such that the complexed G domain and RBD were initially positioned in bulk water. This system was solvated using standard MARTINI water beads with Na^+^ and Cl^−^ ions added to neutralize the excess charges and mimic the physiological ionic strength of 150 mM.

#### Order parameters for defining membrane orientations of RAS

The orientation of the RAS G domain is quantified by its tilt away from the bilayer normal, *θ*_t_, and the azimuthal angle at which that tilt occurs (*i.e*., the rotation), *θ*_r_. Specific definitions of *θ*_t_ and *θ*_r_ are illustrated in Supp. Fig. 7. These definitions are similar to, and inspired by, the definitions of Im and colleagues^54^. Values of *θ*_t_ are computed with respect to a reference orientation, *X*_R_, in which the long axis of C_α_ atoms in RAS helix 5 (H5; V152-K165) from chain A of PDB ID: 4OBE^55^ lies along the global bilayer normal (Cartesian **z** axis) with the G domain’s HVR attachment point directed toward the bilayer (*i.e*., the bilayer is closer to H5′s C-terminal residue K165 than its N-terminal residue V152; Supp. Fig. 7a). For a sampled configuration of RAS, *X*_S_, *R* is the rotation matrix that minimizes the root mean squared deviation between C_α_ atoms of residues 2–26, 40–56, and 69–166 when comparing *RX*_S_ to *X*_R_. Then, *Rz*′ = **z** and *θ*_t_ is the angle between **z** and *z*′ (Supp. Fig. 7b). Conceptualization of *θ*_t_ is facilitated by the fact that *z*′ is roughly parallel to the long axis of the C_α_ atoms in H5. Nevertheless, the approach outlined above (*i.e*., determination of *θ*_t_ based on 140 collectively moving residues in the RAS G domain rather than 14 residues in H5) is adopted to reduce the impact of local conformational fluctuations in H5 on *θ*_t_. The azimuthal angle at which the RAS G domain tilts (*i.e*., the rotation), *θ*_r_, is defined with respect to the center of mass of C_α_ atoms in H2 (S65-T74), H2_COM_. Specifically, the reference frame is translated such that *z*′ passes through the center of mass of C_α_ atoms in H5, H5_COM_, and we define a vector, *a*, that is orthogonal to *z*′, from which it runs to H2_COM_. The vector *p* is the projection of **z** onto the plane *S* that is perpendicular to *z*′ and contains H2_COM_ (*i.e*., the plane *S* contains all points *r* that satisfy *z*′•[*r*-H2_COM_] = 0). Finally, *θ*_r_ is the angle between *p* and *a*, evaluated such that *θ*_r_ > 0° for counter-clockwise rotation from *a* to *p* when viewed from the positive end of *z*′ toward the plane *S* (Supp. Fig. 7b). For reference, Supp. Fig. 7c provides a map of *θ*_r_ values when the RAS G domain is tilted away from the reference orientation in various directions.

#### AA models of the RAS/RBD/CRD ternary complex from computational docking

During docking, we used the aforementioned homology model of BRAF-CRD as the ‘ligand’ and the homology model of KRAS4b/BRAF-RBD as the ‘receptor’ after removing the RAS HVR (residues 171–185). Computational rigid-body docking of this ‘receptor’ and ‘ligand’ was performed using ClusPro^56,57^ with default parameters. The resulting poses for the ternary complex between RAS/RBD/CRD were then filtered based on three criteria: (i) the two hydrophobic loops of CRD must be accessible to allow for CRD membrane anchoring, (ii) the C-terminus of the RBD must be able to connect to the N-terminus of the CRD via a short 4-residue linker, and (iii) the CRD must contact the surface of RAS containing residues N26 and V45. Solution systems were constructed for ternary complex models that remained after applying these filters. For each solvated model, the farnesylated HVR of RAS was replaced with an N-methyl capping group on residue 170. The crystallographic Gpp-NHp molecule bound to RAS was replaced with GTP. Each system was solvated with TIP3P water molecules^48^ in a rhombic dodecahedral box, with a minimum distance of 1.2 nm from any protein atom to any edge of the unit cell. As described in the main text, only the first of three ternary complex models was evaluated beyond this stage. To build the membrane-anchored ternary complex system, a model of the HVR obtained via simulated annealing (PDB 2MSD)^39^ was attached to the G domain using UCSF Chimera^58^. The HVR C-terminal cysteine residue was side chain farnesylated and backbone methylated. A membrane bilayer patch containing 256 lipids per leaflet and composed of 70%:30% POPC:POPS was built using the Membrane Builder module of CHARMM-GUI^49,50^. The ternary complex was anchored to this membrane patch such that both hydrophobic loops of CRD were embedded up to the phosphate layer of the proximal leaflet while the G domain was touching the lipid head groups but did penetrate into the membrane. The farnesyl group was inserted into the hydrophobic core of the membrane. TIP3P water molecules^48^ were then added, along with K^+^ and Cl^–^ ions to neutralize the system charge and to reach a physiological ionic strength of 150 mM. To investigate whether the CRD spontaneously associates with RAS/RBD at the membrane, another version of this membrane-anchored model was built with CRD positioned ~2 nm away from RAS/RBD in the plane of the membrane while the CRD remained attached to the RBD via the native 4-residue linker.

#### CG models of the RAS/RBD/CRD ternary complex

The AA membrane-anchored model of the ternary complex was transformed to CG beads using the MARTINI 2.2 force field^51^ in order to study how this model associates with the membrane. To avoid large structural changes in the ternary complex, an elastic network^52^ connecting the CG beads within RAS, within RBD, within CRD, and between RAS/RBD/CRD were incorporated (excluding residues 171–185 of the RAS HVR). The version of the AA membrane-anchored ternary complex model where the CRD was positioned ~2 nm away from RAS/RBD was also transformed to a CG beads. In this case, an elastic network^52^ connecting the CG beads within RAS, within RBD, within CRD, and between RAS/RBD (but not between RAS/CRD and RBD/CRD) were incorporated (excluding residues 171–185 of the RAS HVR). Both systems were solvated using standard MARTINI water beads. Na^+^ and Cl^–^ ions were added to neutralize the excess charge and mimic the physiological ionic strength of 150 mM.

### Molecular dynamics (MD) protocols

#### CG equilibration of AA membrane-anchored models

Prior to data collection for each membrane-anchored model (CRD-only or ternary complex), we first performed extensive equilibration of the lipid bilayer in the presence of the protein. Each lipid-protein system was converted to a CG representation using the MARTINI 2.2 force field^51^. As MARTINI does not currently contain parameters for handling GTP and divalent ions, the GTP and magnesium ion bound to the RAS and the two zinc ions bound to CRD were removed before CG conversion. For the CRD-only model, the CRD N- and C-termini were modeled with neutral backbones. For the ternary complex model, the RBD N-terminus and CRD C-terminus backbones were also neutral, whereas the RAS N-terminus carried a backbone charge of +1. After CG conversion of each lipid-protein system, solvation was accomplished by adding water beads of which 10% were antifreeze particles that prevent spurious freezing. All protein beads were position-restrained in these equilibration runs, except for the farnesyl group attached to the C-terminus of RAS HVR that was free to move. Equilibration runs were performed for 30 µs.

#### Backmapping of equilibrated membrane-anchored models

The final snapshot after CG equilibration for each membrane-anchored system was used for subsequent all-atom MD simulations. Each MARTINI CG snapshot was converted to an all-atom representation with the CHARMM36 force field^45,46^ via a backmapping protocol that consists of geometric projection of CG beads to atoms followed by system relaxation^59^. Backmapping was performed only for the membrane bilayer lipids, solvent molecules, and the membrane-anchored farnesyl group from KRAS4B, as we have observed random chirality and cis bond errors during backmapping of protein beads. For the latter, the starting all-atom protein conformations (before CG conversion) were instead placed back into the backmapped systems since strong position restraints were applied to all protein beads during the CG equilibration. This also allowed for straightforward re-insertion of GTP, magnesium, and zinc into the backmapped systems. Acetyl and N-methyl caps were added to the CRD N- and C-termini for the CRD-only model, and to the RBD N-terminus and CRD C-terminus for the ternary complex model. Solvent molecules were represented using the TIP3P water model^48^. Monovalent K^+^ and Cl^–^ ions were added to each system to both neutralize the system charge and to reach an ionic strength of 150 mM.

#### AA simulations

The AMBER MD engine (version 16) that has been GPU-optimized for simulating explicit solvent systems^60,61^ was used for running all atomistic simulations. Particle mesh Ewald (PME) electrostatics^62^ were used along with Coulomb and Lennard Jones cut-offs of 1.2 nm and potential switching at 1.0 nm. Constant temperature was maintained at 310 K via Langevin dynamics^63^ with a collision frequency of 1.0 ps^–1^. Semi-isotropic pressure coupling was set for each system at 1 bar using a Monte Carlo barostat^64^ with a relaxation time of 4.0 ps. Bonds containing hydrogen atoms were constrained using the SHAKE algorithm^65^. A hydrogen mass repartitioning approach^66^ allowed the use of a 4-fs time step, and each system was simulated for a total of 1 µs. Data collection and analyses was performed on the last 2/3 of each simulation.

#### CG simulations

The GROMACS MD engine (version 5.1.3)^67^ was used in combination with the MARTINI 2.2 force field^51^ for running CG simulations. We followed a recent update in CG parameters set-up for performing the simulations^68^. Simulations used a 30 fs time-step. Reaction-field electrostatics^69^ was used with a Coulomb cut-off of 1.1 nm and dielectric constants of 15 or 0 within or beyond this cut-off, respectively. A cut-off of 1.1 nm was also used for calculating Lennard Jones interactions, using a scheme that shifts the Van der Waals potential to zero at this cut-off. Constant temperature was maintained at 310 K via separate coupling of the solvent and membrane/protein components to velocity rescaling thermostat^70^ with a relaxation time of 1.0 ps. Semi-isotropic pressure coupling was set for each system at 1 bar using a Berendsen barostat^71^ with a relaxation time of 12.0 ps. Each system was replicated 10 times, with 200 µs per replica collected for the CRD simulations and 100 µs per replica for the RAS/RBD and RAS/RBD/CRD simulations.

### Experimental procedures

#### Expression and purification of RAF-CRD

*E. coli* strain BL21 STAR (*rne*131) containing the DE3 lysogen and rare tRNAs (pRare plasmid) was transformed with the expression plasmid (His6-MBP-tev-RAF1CRD(136–188)). The *E. coli* seed culture was inoculated from a glycerol stock of the transformed strain and grown in 50 mL of MDAG medium^72^ in a 250 mL baffled shakeflask for 16 hours at 37 °C until mid-log phase growth. 2% (1:50 of production volume) or 40 mL was removed and centrifuged at 3000x g for 10 minutes at 25 °C and the pellet was resuspended into 40 mL of Mod M9 medium containing 2 g/L glucose (for ^13^C labelled growths, ^13^C Glucose), 1 g/L NH_4_Cl (for ^15^N labelled growths ^15^N NH_4_Cl), 2 mM MgSO_4,_ 100 µM CaCl_2,_ 4 µM ZnSO_4_, 1 µM MnSO_4_, 4.7 µM H_3_BO_3_ and 0.7 µM CuSO_4._ The suspension was used to inoculate 2 L of Mod M9 in a 3-liter Bioflow 110 bioreactor (Eppendorf/NBS). The culture was grown at 37 °C with the airflow set at 2.0 LPM, while the agitation was maintained at 481 RPM. When the OD600 reached 0.4–0.6 (~3 hours) ZnCl_2_ was added to a final concentration of 300 µM and IPTG was added to a final concentration of 500 µM. After 3 additional hours of growth the cells were collected by centrifugation using a Beckman Coulter Avanti J-20 XP and JLA 8.1 rotor at 5000x g. Cell pellets were immediately frozen at −80 °C. Proteins were essentially purified as by Gillette and colleagues^73^, with the following modifications: MgCl_2_ was not added to purification buffers, cells were resuspended at a ratio of 20 mL buffer/1000 OD_600_ units, the homogenized cells were lysed using a Microfluidizer M-110-EH (Microfluidics Corp., Westwood, MA) at 10000 psi for 2 passes, the IMAC pools were taken directly to TEV digestion/dialysis (omitting the lower pH dialysis and IEX steps), and the proteins were buffer exchanged to final buffers as by Dharmaiah and colleagues^74^, using 20 mM Hepes, pH 7.3, 150 mM NaCl, 1 mM TCEP for RAF1 CRD and 20 mM Hepes, pH 7.3, 100 mM NaCl, 0.5 mM EDTA for pMSP delH5.

#### Expression and purification of pMSP delH5

The membrane scaffold protein (MSP) ΔH5 clone was obtained from the group of Gerhard Wagner at Harvard University^75^. Similar methods were used for the transformation of pMSP delH5. A glycerol stock was used to inoculate 300 ml of MDAG medium in a 2 L baffled shakeflask for 16 hours at 37 °C until mid-log phase growth. 2% (1:50 of production volume) or 300 mL was used to inoculate 15 L of Terrific Broth in a 20-liter Bioflow IV bioreactor (Eppendorf/NBS). The culture was grown at 37 °C with the airflow set at 15.0 LPM, while the agitation was maintained at 350 RPM. When the OD600 reached 3.5 (~4 hours) IPTG was added to a final concentration of 500 µM. After 3 additional hours of growth the cells were collected and stored as detailed above. Cells were thawed and homogenized with buffer A (20 mM HEPES, pH 7.3, 300 mM NaCl, 1 mM TCEP, 50 mM imidazole, and 1:1000 v/v protease inhibitor, Sigma-Aldrich P-8849) and lysed and clarified as outlined above for RAF1 CRD. Clarified lysate was applied to a 200 ml IMAC column (Ni SepharoseTM High Performance, GE Healthcare) equilibrated with buffer A. After washing the column with buffer A to baseline UV_280_, proteins were eluted from the column with a gradient elution of 5 column volumes from 10–500 mM imidazole in buffer A. Fractions containing the target protein were pooled, concentrated, and dialyzed overnight at 4 °C to a final buffer of 20 mM Tris-HCl, pH 7.5, 10 mM NaCl, and 0.5 mM EDTA. A slight precipitate was removed by centrifugation (20 min at 4 °C, 5000x g), the sample filtered using a 0.22 micron syringe filter, 1.0 ml aliquots snap-frozen in liquid nitrogen, and the final samples stored at −80 °C. Final protein concentration was 5.7 mg/ml (A280).

#### Preparation of nanodiscs

POPC and POPS lipids were purchased from Avanti Polar Lipids in chloroform with concentrations determined by total phosphate analysis. Nanodiscs containing 100% POPC and 70% POPC/30% POPS were prepared based on a published protocol with minor modifications^76^. Briefly, stock lipids were mixed together at room temperature, slowly dried using argon gas while incubating in a bead heat bath at 37 °C, and then put on a vacuum lyophilyzer overnight to remove all residual organic solvents. The dried lipids were reconstituted to 65 mM with 130 mM cholate (Sigma -Aldrich) in 20 mM HEPES pH 7.4, 100 mM NaCl, and 0.5 mM EDTA buffer, and mixed with MSP ΔH5 in a 30:1 lipid to protein ratio. The mixtures were mixed on a Nutator at 4 °C for 1 hour, and then 0.6 g/mL of washed bio-beads (Bio-Rad laboratory) were added and incubated at 4 °C for additional 4.5 hours. Afterwards, the nanodiscs were removed from the bio-beads by careful pipetting and purified using gel filtration chromatography on an AKTA FPLC with a GE Superdex 200 increase column (10 × 300 mm) with the mobile buffer containing 20 mM HEPES pH 7.4, 100 mM NaCl, and 0.5 mM EDTA at 0.5 mL/min flow rate. Peak fractions were pooled and analyzed by dynamic light scattering to assess homogeneity; pooled fractions consistently had a polydispersity of less than 15% and diameter of ~7.2–7.5 Å.

#### NMR experiments

NMR data were collected on an Agilent 800 MHz and Bruker 600 & 900 MHz spectrometers at 25 °C. Data were processed with NMRPipe^77^ and analyzed using NMRFAM-SPARK^78^. The CRD backbone assignments were obtained from HNCACB and CBCACONH on a 0.5 mM ^13^C/^15^N-CRD(G,136–188) sample. Backbone assignments were obtained for 51 of the 54 expected peaks; NH peaks were not observed for L136 and H175 as well as the extra glycine at the N-terminal. The peak labeled as uk1 is probably W187′s sidechain NH. 800 MHz TROSY spectra were collected for ^15^N-CRD (131 µM), ^15^N-CRD: 100% POPC nanodiscs (188 µM), and ^15^N-CRD:70% POPC-30% POPS nanodiscs (188 µM) using 128 increments, 256 scans, 39.3 ms acquisition time, and 1s relaxation delay. Spectra were acquired with 128 increments, 64 scans for free CRD and 256 scans for CRD-nanodiscs, 80 ms acquisition time, and 0.3s relaxation delay. The buffer was 20 mM HEPES, pH 7.4, 150 mM NaCl, 1 mM TCEP, 7% D_2_O and 6% sodium azide for all samples. Chemical shift perturbations were quantified using the following equation:

1 $${\rm{CSP}}({\rm{ppm}})=\sqrt{\frac{{({\Delta }H)}^{2}+{({\Delta }N)}^{2}/25}{2}}$$

## Electronic supplementary material

Supporting Information
