# Supplementary material for: Molecular recognition of RAS/RAF complex at the membrane: Role of RAF cysteine-rich domain
Source: Sci Rep. 2018 May 31;8:8461. doi: 10.1038/s41598-018-26832-4 (PMC5981303; doi:10.1038/s41598-018-26832-4)
Supplement: Supplementary file 1 — Supporting Information [file 41598_2018_26832_MOESM1_ESM.pdf]

Supporting information

Molecular recognition of RAS/RAF complex at the membrane: Role of RAF cysteine-rich domain

Timothy Travers, Cesar A. López, Que N. Van, Chris Neale, Marco Tonelli, Andrew G. Stephen, S. Gnanakaran

Supporting Methods

POPC, POPS, and farnesyl parameterization strategy. To maximize the compatibility of our parameters with those of other molecules available in the Martini force field [1], we followed the basic philosophy inherent to that model [2]. For POPC/POPS, the CG bead types and their respective non-bonded terms were not modified from the original lipid set (see **Parameters** section below). The farnesyl group, however, required a careful selection of bead types based on its octanol/water partition coefficients, in line with the original parametrization approach [1]. Our strategy also involved modifying the bonded terms (bonds and angles) for POPC/POPS and farnesyl such that their sampled populations best fit the corresponding distributions obtained from a pseudo-CG mapping obtained from AA simulations using the CHARMM36 force field [3]. To this end, the AA trajectories were converted into pseudo-CG trajectories using a geometrical projection method [4]. Distributions of sampled bond and angle terms were then collected from these pseudo-CG trajectories and used as target functions in the iterative modification of Martini bonded parameters until a close match was achieved.

To determine the accuracy of the updated POPC/POPS CG parameters, we first compared the electron density profiles for both lipids using CHARMM36, the standard Martini V2.2 lipid parameters, and the optimized Martini set (**Supp. Fig. 24**). We observed a good agreement between the three sets, although the optimized Martini set approaches much better the profiles obtained using CHARMM36. As a second test, we also measured the area per lipid (APL) for both lipids based on CG simulations using either the standard or optimized Martini parameters, and compared these with experimentally measured values. For POPC, the simulated APL was 0.65 ± 0.0075 nm^2 and 0.64 ± 0.0062 nm^2 for standard and optimized Martini, respectively, which both correspond well with the experimental value of 0.64 nm^2 [5]. For POPS, we found that the simulated APL using optimized Martini (0.60 ± 0.0062 nm^2) was closer than that using standard Martini (0.64 ± 0.0069 nm^2) to the experimental value of 0.55 nm^2 [6].

The atomistic representation of the farnesyl group, in combination with its respective Martini representation, is shown in **Supp. Fig. 25a**. To assess the accuracy of the CG farnesyl parameters, the PMF for membrane permeation was computed and compared between AA umbrella sampling

runs and CG simulations (umbrella sampling and parallel tempering metadynamics) (**Supp. Fig. 25a**, top-right plot). We found that these computed PMFs were in very good agreement with each other. Although the independent solvation free energies obtained from AA and CG runs are not in total agreement, the calculated octanol-water partition coefficients match very well (**Supp. Fig. 25a**, bottom table). The preferential localization of farnesyl in a pure POPC bilayer was also tested, and **Supp. Fig. 25b** shows that the resulting electron density profiles match quite well between AA and CG.

CG reparametrization simulations. Initial configurations of CG membrane patches with homogeneous lipids were obtained by CG-mapping the final snapshots of POPC and POPS from AA simulations. The resulting CG systems contained 64 lipids per leaflet, 1600 Martini water beads (50 atomistic water molecules per lipid) and 150 mM NaCl to preserve the ionic strength from AA simulations. For the parametrization of the farnesyl group, a cubic box containing 300 atomistic farnesyl molecules was run and pseudo-CG transformed for iterative fitting to Martini. Simulations were run with GROMACS version 5.1.2 [7] in combination with the Martini 2.2 force field [1]. We followed a recent update in setting up the MD parameters file for performing the CG simulations [8]. The simulation time step was set at 30 fs. Reaction-field electrostatics [9] were used with a Coulomb cut-off of 1.1 nm and dielectric constants of 15 or 0 when within or beyond this cut-off, respectively. Lennard-Jones interactions were cut off at 1.1 nm, where the potential was shifted to zero. Constant temperature was maintained at 310 K via separate coupling of the solvent (water and ions) and lipid components to velocity-rescaling thermostats [10] with relaxation times of 1.0 ps. During equilibration, the pressure was semi-isotropically coupled at 1 bar using Berendsen barostats [11] with relaxation times of 12.0 ps and compressibilities of $3x10^{-4}$ bar^{-1}. After equilibration, Parrinello-Rahman barostats [12] were then used. Analyses were performed using either in-house scripts or tools provided by the GROMACS package.

Supporting References:

1. Marrink, S. J., Risselada, H. J., Yefimov, S., Tieleman, D. P., & de Vries, A. H. The MARTINI force field: coarse grained model for biomolecular simulations. *J Phys Chem B*. **111**, 7812–7824 (2007).
2. Marrink, S. J. & Tieleman, D. P. Perspective on the Martini model. *Chem Soc Rev*. **42**, 6801–6822 (2013).
3. Klauda, J. B. *et al.* Update of the CHARMM all-atom additive force field for lipids: validation on six lipid types. *J. Phys. Chem. B.* **114**, 7830-7843, (2010).
4. Wassenaar, T. A. & Pluhackova, K. Going backward: A flexible geometric approach to reverse transformation from coarse grained to atomistic models. *J Chem Theory Comput*. **10**, 676-690 (2013).
5. Kucerka, N., Nieh, M. P., & Katsaras, J. Fluid phase lipid areas and bilayer thickness of commonly used phosphatidylcholines as a function of temperature. *Biochim Biophys Acta.* **1808**, 2761-2771 (2011).
6. Mukhopadhyay, P., Monticelli, L., & Tieleman D. P. Molecular dynamics simulation of a palmitoyl-oleoyl phophatidylserine bilayer with Na^+ counterions and NaCl. *Biophys J.* **86**, 1601-1609 (2004).
7. Páll, S., Abraham, M. J., Kutzner, C., Hess, B. & Lindahl, E. in Solving Software Challenges for Exascale (eds. Markidis, S. & Laure, E.) 8759, 3–27 (Springer International Publishing, 2014).
8. de Jong, D. H., Baoukina, S., Ingólfsson, H. I., & Marrink, S. J. Martini straight: Boosting performance using a shorter cutoff and GPUs. *Comp Phys Comm*. **199**, 1–7 (2016).
9. Tironi, I. G., Sperb, R., Smith, P. E., & van Gunsteren, W. F. A generalized reaction field method for molecular dynamics simulations. *J Chem Phys*. **102**, 5451 (1995).
10. Bussi, G., Donadio, D. & Parrinello, M. Canonical sampling through velocity rescaling. *J Chem Phys*. **126**, 014101 (2007).
11. Berendsen, H. J. C., Postma, J. P. M., van Gunsteren, W. F., DiNola, A., & Haak, J. R. Molecular dynamics with coupling to an external bath. *J Chem Phys*. **81**, 3684 (1984).
12. Nosé, S. & Klein, M. L. Constant pressure molecular dynamics for molecular systems. *Mol Phys*. **50**, 1055-1076 (1983).

Parameters

```
[ moleculetype ]
; Name            nrexcl
POPC              1

[ atoms ]
;   nr      type  resnr residue  atom   cgnr     charge       mass  typeB    chargeB      massB
    1        Q0    1    POPC   NC3     1        1       72.0000
    2        Qa    1    POPC   PO4     2       -1       72.0000
    3        Na    1    POPC   GL1     3        0       72.0000
    4        Na    1    POPC   GL2     4        0       72.0000
    5        C1    1    POPC   C1A     5        0       72.0000
    6        C3    1    POPC   D2A     6        0       72.0000
    7        C1    1    POPC   C3A     7        0       72.0000
    8        C1    1    POPC   C4A     8        0       72.0000
    9        C1    1    POPC   C1B     9        0       72.0000
   10        C1    1    POPC   C2B    10        0       72.0000
   11        C1    1    POPC   C3B    11        0       72.0000
   12        C1    1    POPC   C4B    12        0       72.0000

[ bonds ]
1    2    1    0.40  9000
2    3    1    0.42  2000
3    4    1    0.312 3000
3    5    1    0.55  2000
5    6    1    0.55  3800
6    7    1    0.55  1200
7    8    1    0.55  2000
4    9    1    0.47  3600; 0.3
9   10    1    0.47  3800
10  11    1    0.47  3800
```

```
11  12    1    0.47  3600

[ angles ]
 1  2  3 2 144.77 20.98
 2  3  5 2 145.00 25.00
 2  3  4 2 110.00 25.00
 5  3  4 2   82.00 10.00
 3  5  6 2 180.00 25.00
 5  6  7 2 130.00 35.00
 6  7  8 2 130.00 35.00
 3  4  9 2 123.53 42.25
 4  9 10  2 152.89 32.55
 9 10 11  2 180.00 35.00
10 11 12  2 180.00 35.00
```

[moleculetype]

; Name nrexcl

POPS 1

[atoms]

; nr	type	resnr	residue	atom	cgnr	charge	mass	typeB	chargeB	massB
1	P5	1	POPS	CNO	1	0	72.0000			
2	Qa	1	POPS	PO4	2	-1	72.0000			
3	Na	1	POPS	GL1	3	0	72.0000			
4	Na	1	POPS	GL2	4	0	72.0000			
5	C1	1	POPS	C1A	5	0	72.0000			
6	C3	1	POPS	D2A	6	0	72.0000			
7	C1	1	POPS	C3A	7	0	72.0000			
8	C1	1	POPS	C4A	8	0	72.0000			
9	C1	1	POPS	C1B	9	0	72.0000			
10	C1	1	POPS	C2B	10	0	72.0000			
11	C1	1	POPS	C3B	11	0	72.0000			
12	C1	1	POPS	C4B	12	0	72.0000			

[bonds]

1 2 1 0.40 9000

2 3 1 0.42 2000

3 4 1 0.312 3000

3 5 1 0.55 2000

5 6 1 0.55 3800

6 7 1 0.55 1200

7 8 1 0.55 2000

4 9 1 0.47 3600; 0.3

9 10 1 0.47 3800

10 11 1 0.47 3800

11 12 1 0.47 3600

[angles]

1	2	3	2	144.77	20.98
2	3	5	2	145.00	25.00
2	3	4	2	110.00	25.00
5	3	4	2	82.00	10.00
3	5	6	2	180.00	25.00
5	6	7	2	130.00	35.00
6	7	8	2	130.00	35.00
3	4	9	2	123.53	42.25
4	9	10	2	152.89	32.55
9	10	11	2	180.00	35.00
10	11	12	2	180.00	35.00

[moleculetype]

; Name nrexcl

FAR 1

[atoms]

; nr	type	resnr	residue	atom	cgnr	charge	mass	typeB	chargeB	massB
1	SC3	1	FAR	SC2	1	0	72.0000			
2	C2	1	FAR	SC3	2	0	72.0000			
3	SC3	1	FAR	SC4	3	0	72.0000			
4	SC3	1	FAR	SC5	4	0	72.0000			

[bonds]

1 2 1 0.22 15000

2 3 1 0.500 500

3 4 1 0.2170 10000

[angles]

1 2 3 2 115.00 30.00

2 3 4 2 124.50 30.00

Supporting Figures

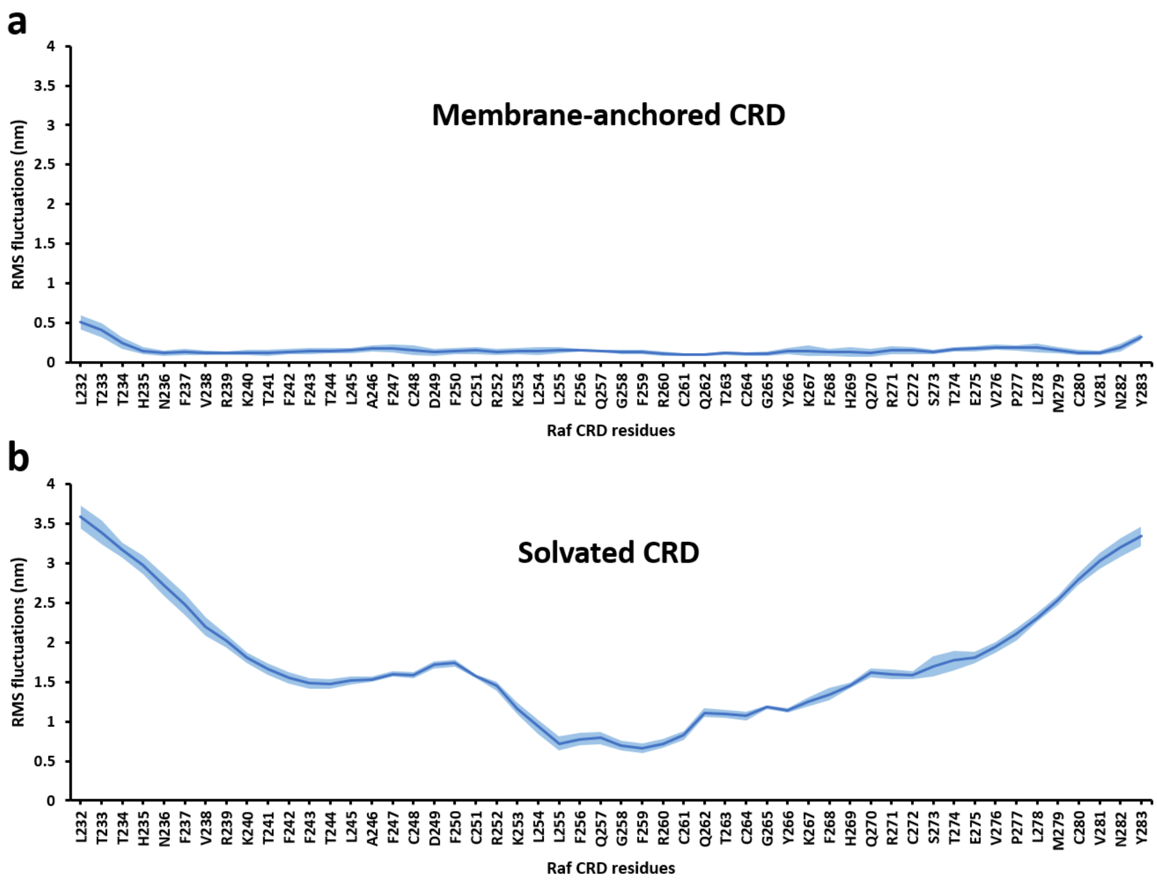

Supp. Figure 1. Per-residue RMSF plots for (a) membrane-anchored and (b) solvated AA simulations of CRD. Light blue error bars give s.e.m. over five simulations.

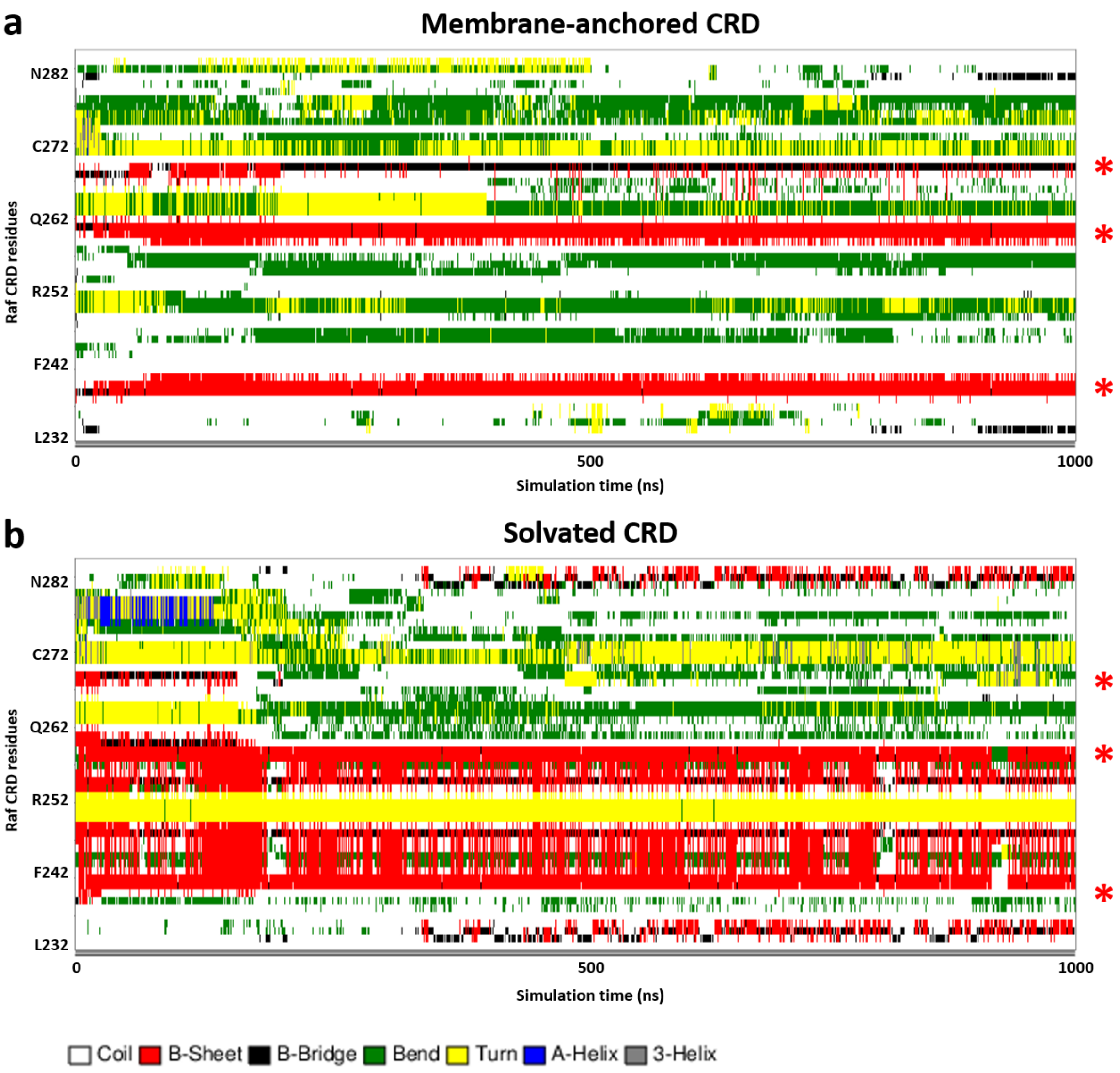

Supp. Figure 2. Secondary structure profiles over simulation time for one of the (a) membrane-anchored and (b) solvated AA simulations of CRD. Color-coding of secondary structural elements are given at the bottom. Red asterisks indicate where the three β-strands comprising the core β-sheet structure are located.

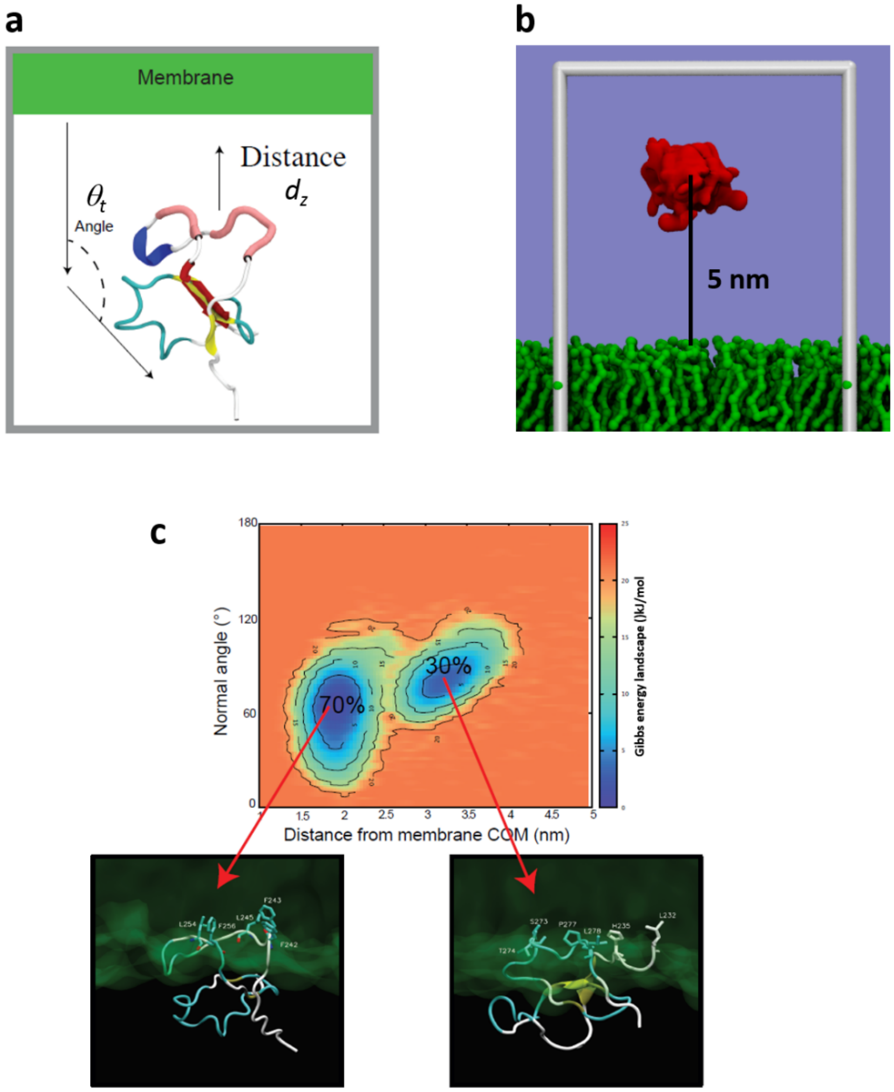

Supp. Figure 3. (a) Distance and angle order parameters used for defining membrane orientations of CRD (see main text for a description of these order parameters). (b) Initial position of CRD away from the membrane in the CG simulations. (c) Free energy (kJ/mol) contour map of membrane-anchored configurations of CRD from CG simulations, using the order parameters shown in (a). The dominant basin (comprising 70% of sampled snapshots) corresponds to the membrane orientation with both CRD hydrophobic loops embedded (bottom-left inset). The other basin (comprising the remaining 30% of sampled snapshots) corresponds to a membrane orientation that has both hydrophobic loops not embedded. We are currently investigating the significance of this second basin.

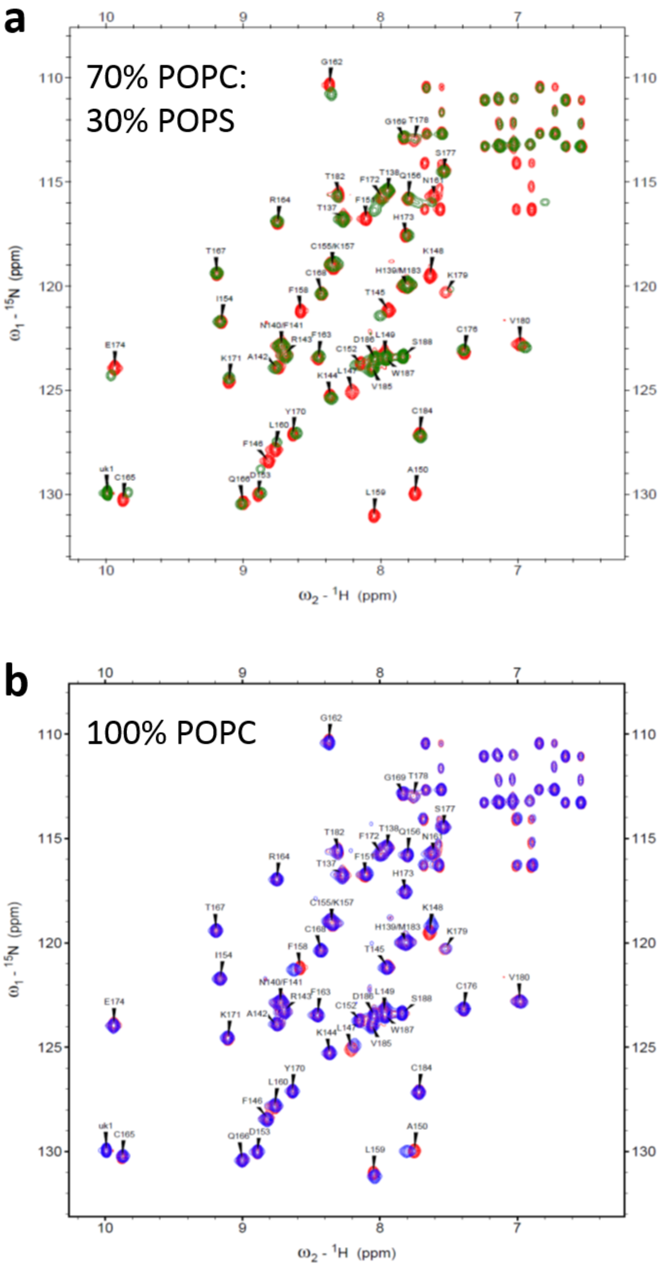

Supp. Figure 4. Comparison of 800 MHz TROSY spectra for: (a) ^{15}N-CRD:70% POPC/30% POPS (green) overlaid on free ^{15}N-CRD (red), and (b) ^{15}N-CRD:100% POPC (blue) overlaid on free ^{15}N-CRD (red).

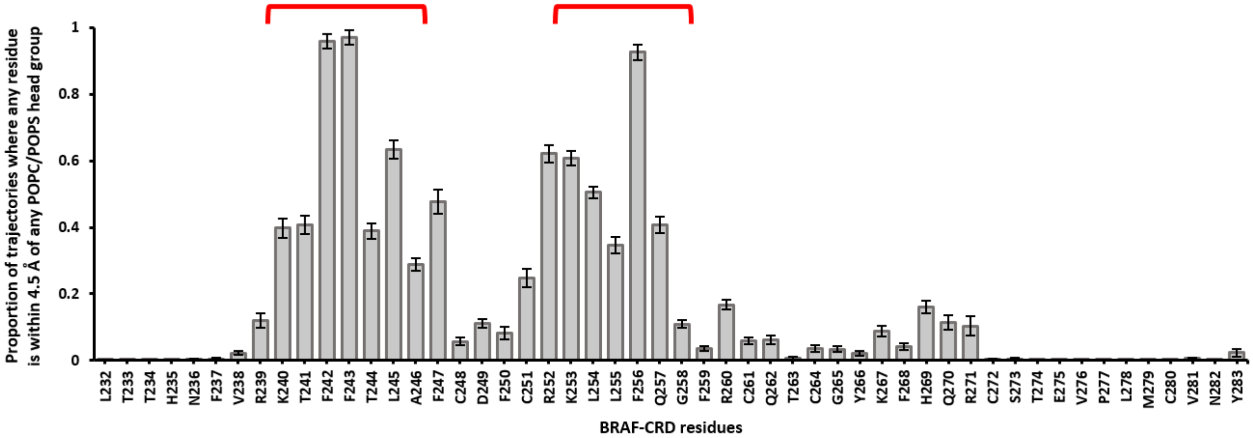

Supp. Figure 5. Per-residue membrane contact profiles for BRAF-CRD from CG simulations, with a higher value indicating a larger proportion of the simulation time in which the residue was making at least one membrane contact. Membrane contacts are defined here as occurring when any residue bead is within 4.5 Å of any lipid head group. Red brackets denote the residues belonging to the two hydrophobic loops of CRD. Error bars give the s.e.m. from ten simulations.

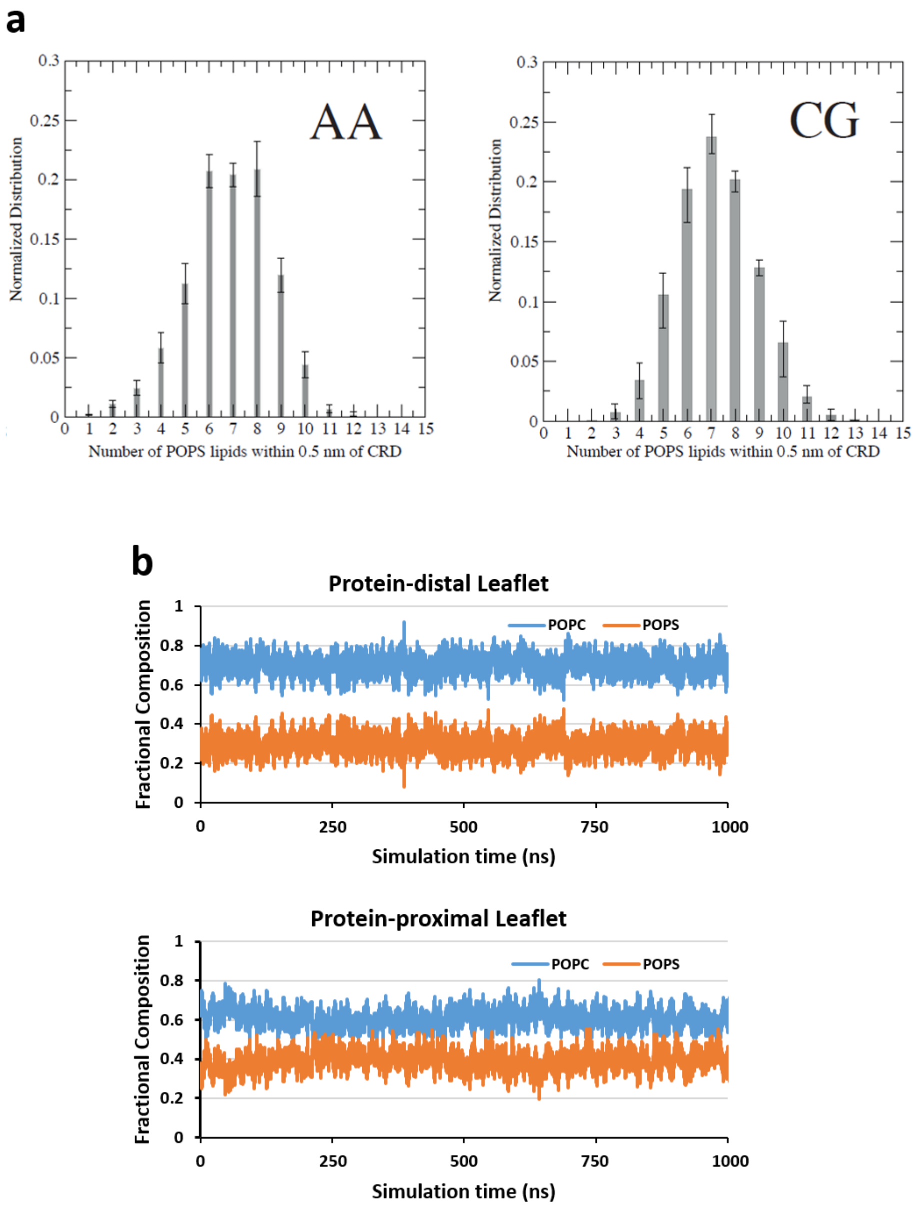

Supp. Figure 6. (a) Normalized distributions of POPS lipid counts around membrane-anchored CRD in AA (left) and CG (right) simulations. Error bars give s.e.m. from five AA simulations (left) or ten CG simulations (right). (b) Time profiles for fractional composition of POPC:POPS within 0.45 nm of membrane-anchored CRD in the protein-distal (top) and protein-proximal (bottom) leaflets from one of the AA simulations. For the protein-distal leaflet, the edges of the CRD were projected to this leaflet along the z-axis in order to measure the fractional compositions.

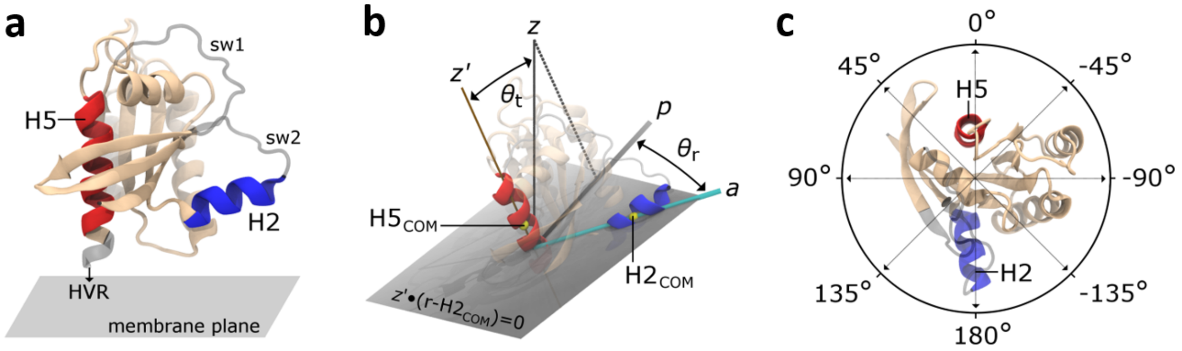

Supp. Figure 7. Quantification of RAS G domain orientation. (a) Reference configuration of the RAS G domain, X_R, with respect to the global membrane plane. H2 is blue, H5 is red, residues 2-26, 40-56, and 69-166 used in the definition of the rotation matrix R are orange, and other residues are gray. Switch 1 (sw1) and 2 (sw2), and the H5 connection to the HVR are labeled. (b) Schematic describing the definitions of θ_t and θ_r. To compute θ_t: **z** is the global bilayer normal; $Rz'=\mathbf{z}$, where R is the rotation matrix that aligns the reference orientation with a sampled configuration; and θ_t is the angle between **z** and z'. To compute θ_r: p is the projection of **z** onto the plane S defined by $z'\bullet(r\text{-H2}_{COM})=0$, where H2_{COM} is the center of mass of C_α atoms in H2; a is the vector in S that runs from z' to H2_{COM}; and θ_r is the angle between p and a, evaluated such that $\theta_r > 0°$ for counter-clockwise rotation from a to p when viewed from the positive end of z' toward the plane S. (c) Values of θ_r obtained for tilts that move the proximal region of the RAS G domain (including sw1) toward the bilayer in various directions, viewed from positive **z** toward the global bilayer plane.

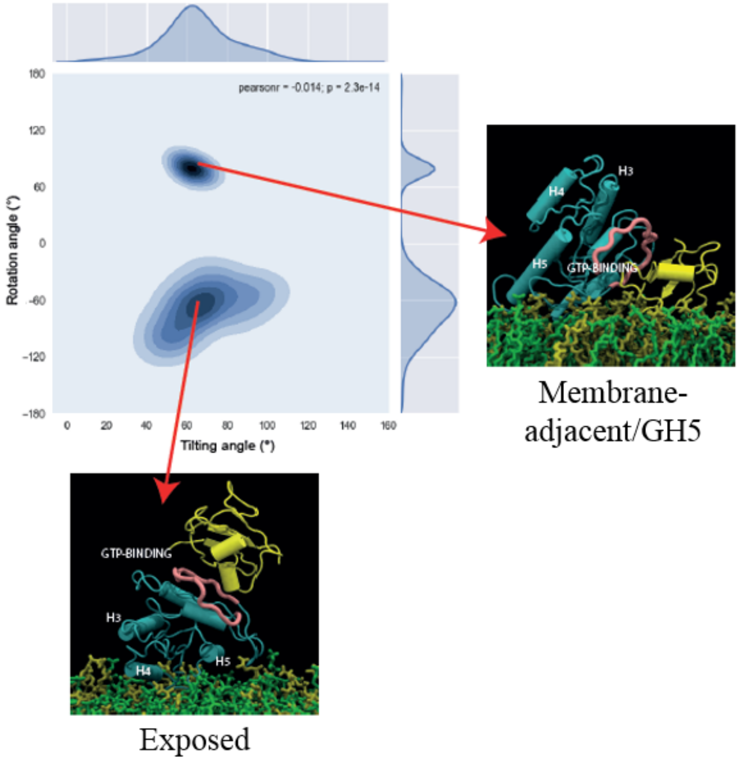

Supp. Figure 8. Population-based contour map showing the Exposed and Membrane-adjacent/GH5 orientations from CG simulations of RAS/RBD, using the order parameters described in **Supp. Figure 7**.

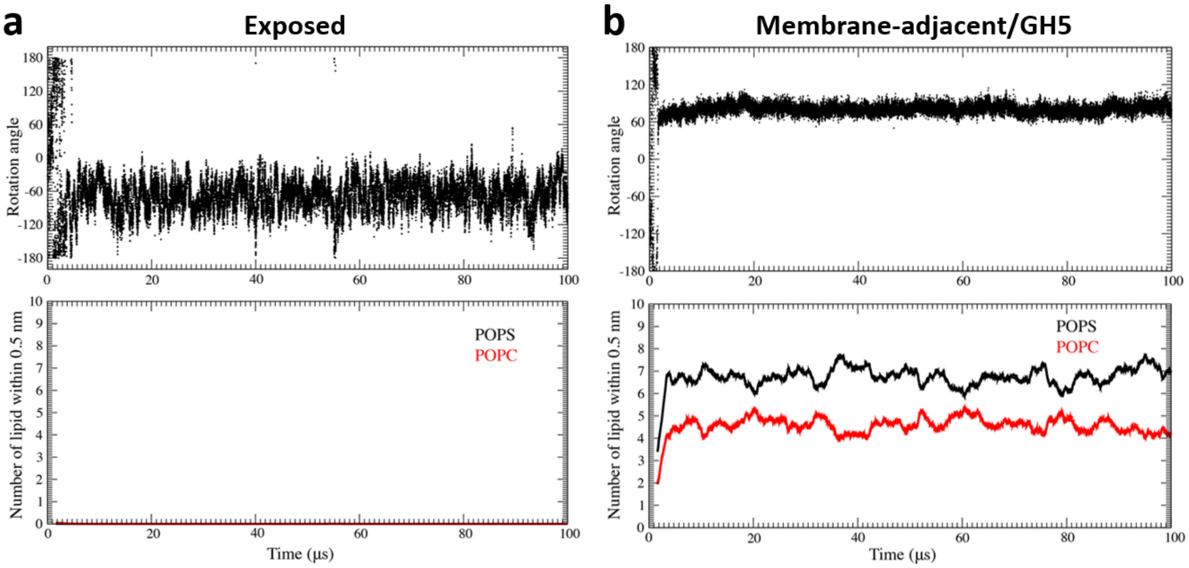

Supp. Figure 9. Time profiles for the rotation angle order parameter (top) and count of POPC/POPS lipid interactions with RBD (bottom) for frames of RAS/RBD CG simulations adopting either the (a) Exposed or the (b) Membrane-adjacent/GH5 orientations.

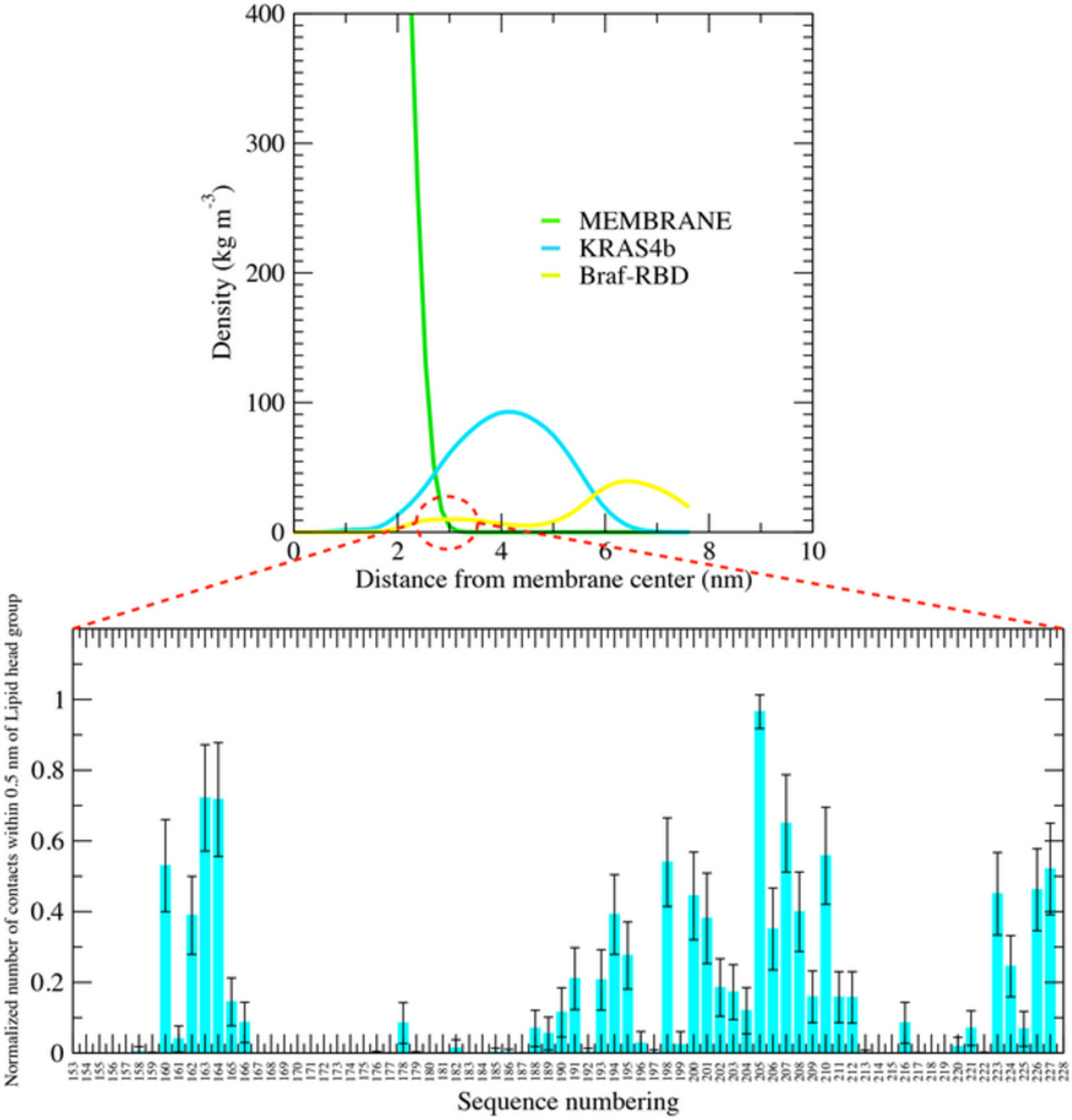

Supp. Figure 10. (top) Mass density profiles for membrane (green), RAS (cyan), and BRAF-RBD (yellow) from the membrane-anchored RAS/RBD CG simulations. The smaller RBD density peak near the membrane corresponds to the Membrane-adjacent/GH5 orientation, while the Exposed orientation comprises the larger RBD density peak. (bottom) Per-residue membrane contact profiles for RBD from CG simulation snapshots adopting the Membrane-adjacent/GH5 orientation.

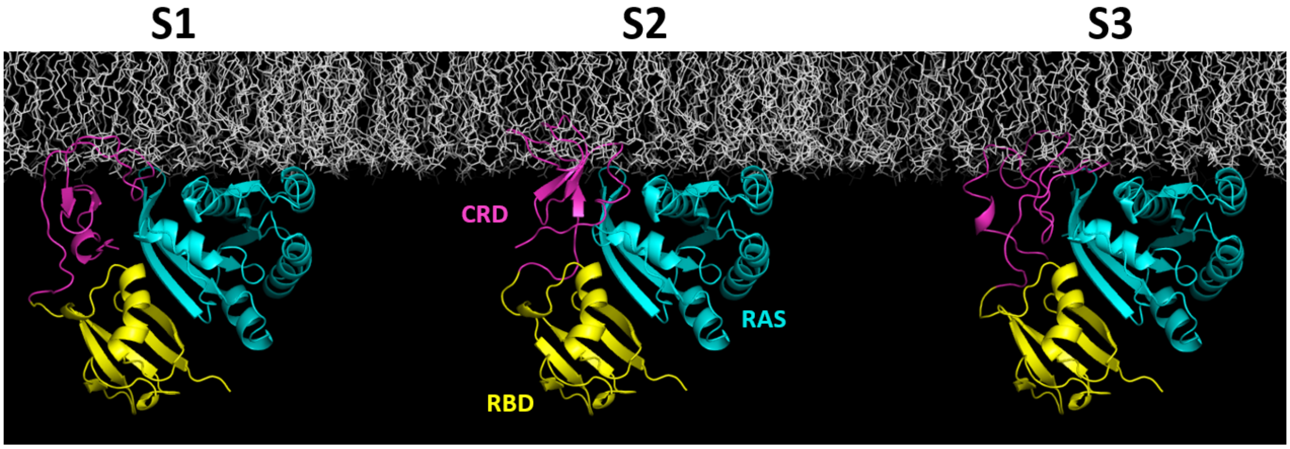

Supp. Figure 11. Three predicted models for the RAS/RBD/CRD ternary complex based on computational docking followed by filtering using criteria based on solution experiments. The three models are depicted here with RAS/RBD in the same orientation, in order to highlight the different orientations of CRD relative to RAS/RBD in the different models. Each model was anchored to the membrane using the two hydrophobic loops of CRD and the farnesylated HVR of RAS.

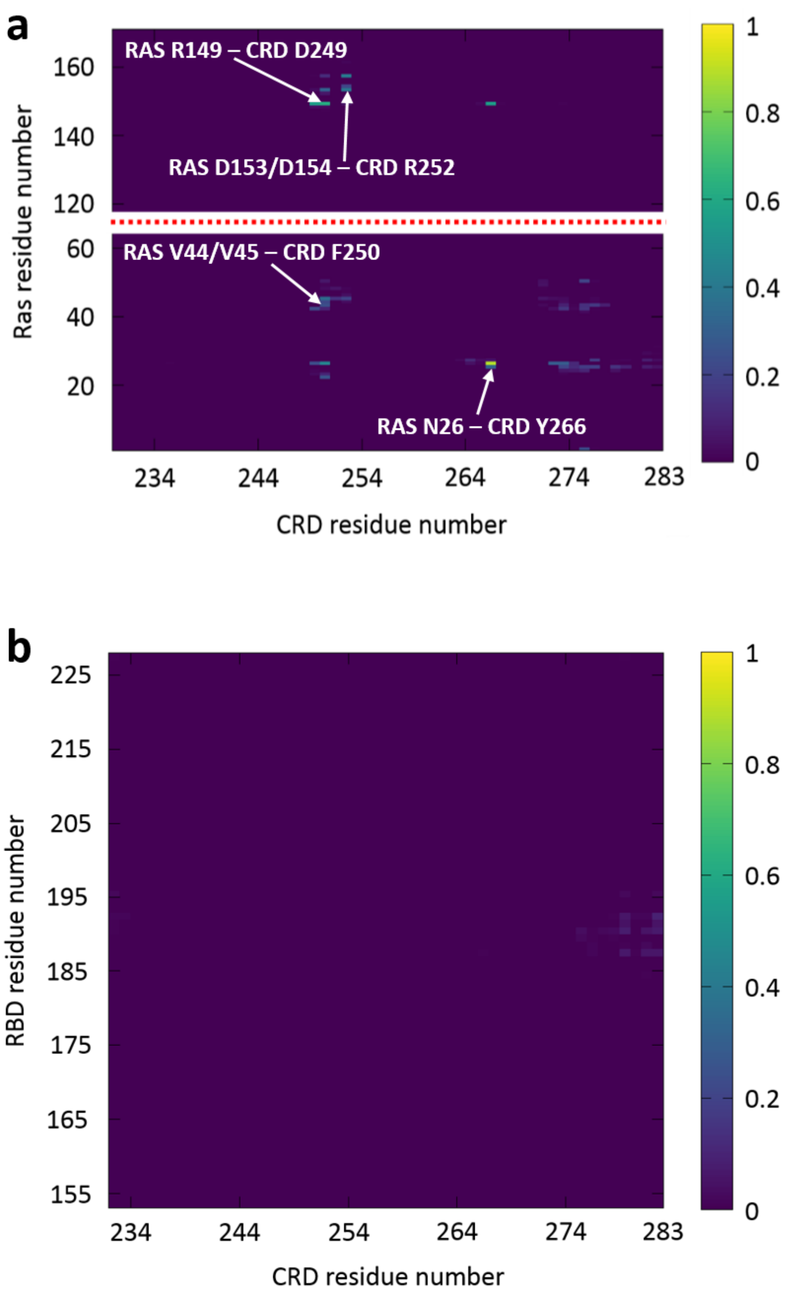

Supp. Figure 12. Residue contact maps between (a) RAS/CRD and (b) RBD/CRD based on five AA simulations of the membrane-anchored docked model S1. White arrows in (a) point to contacts between RAS and CRD that were also identified in the solvated simulations of docked model S1 (see **Figure 4a** in the main text).

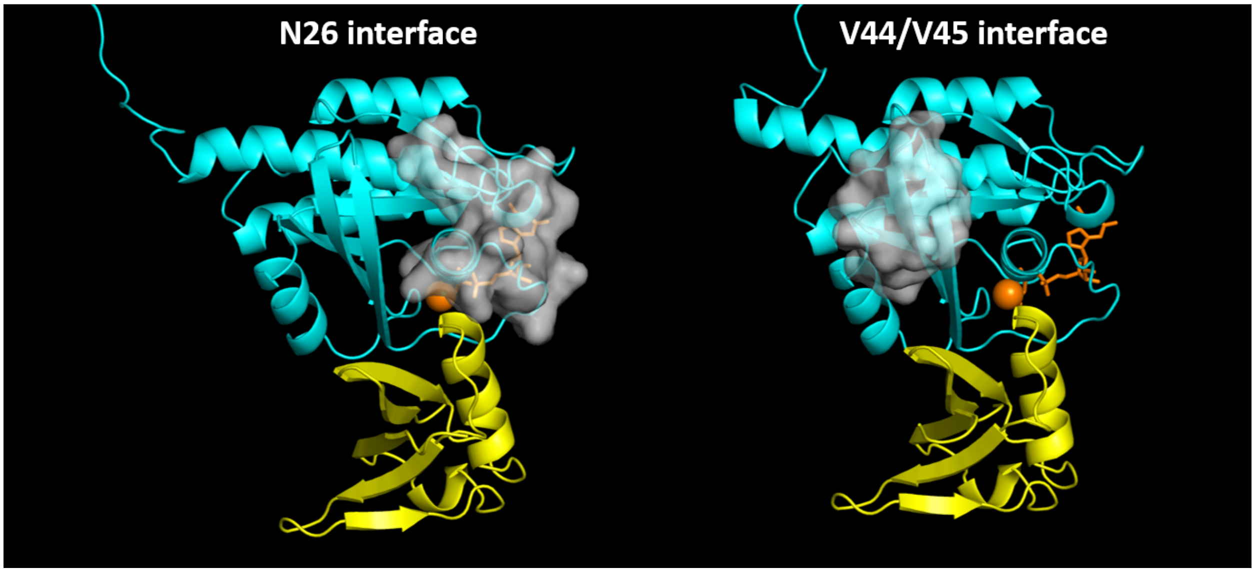

Supp. Figure 13. Translucent white surfaces on RAS (cyan cartoons) show alternate contact surfaces with CRD, particularly for the AA simulations where CRD is in contact either with the interface around N26 only (left) or with the interface around V44/V45 only (right). RBD is shown with yellow cartoons. GTP bound to RAS is shown as orange sticks, along with Mg^{2+} as an orange sphere.

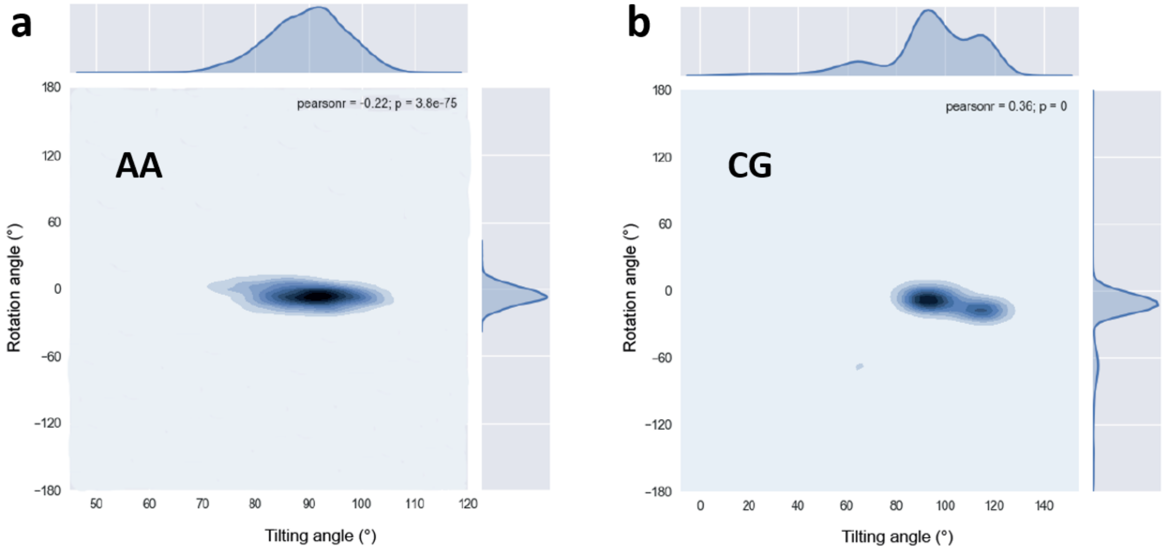

Supp. Figure 14. Population-based contour maps showing the Exposed orientation from (a) AA and (b) CG simulations of membrane-anchored docked model S1 of the ternary complex, using the order parameters described in **Supp. Figure 7**.

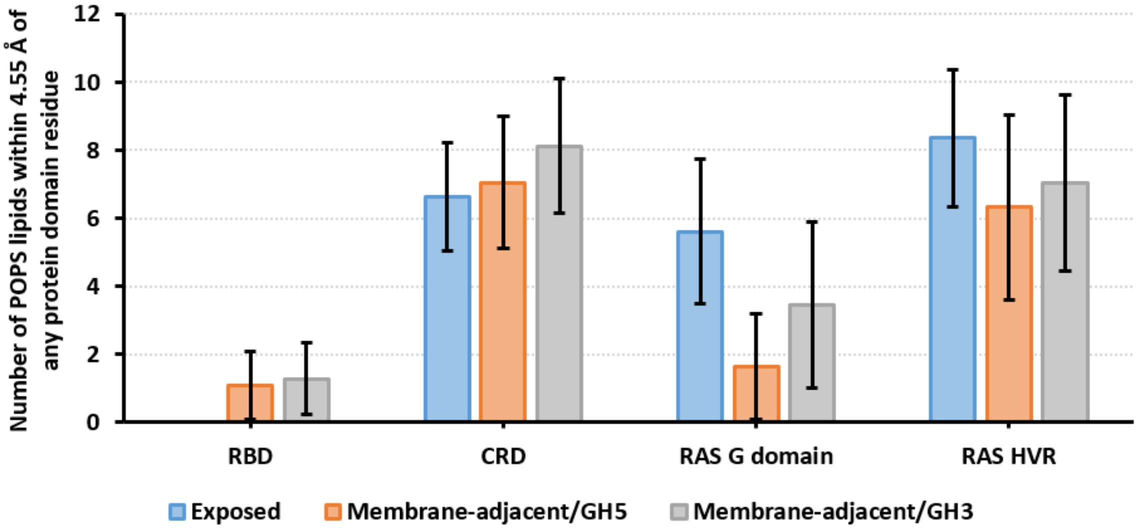

Supp. Figure 15. Average number of POPS lipid around RBD, CRD, G domain, and HVR in the following simulations of the RAS/RBD/CRD ternary complex: (blue) AA simulations of the membrane-anchored docked model S1 that sampled the Exposed RAS orientation, (orange) CG simulations that sampled the Membrane-adjacent/GH5 RAS orientation, and (gray) CG simulations that sampled the Membrane-adjacent/GH3 RAS orientation. Error bars give standard deviation.

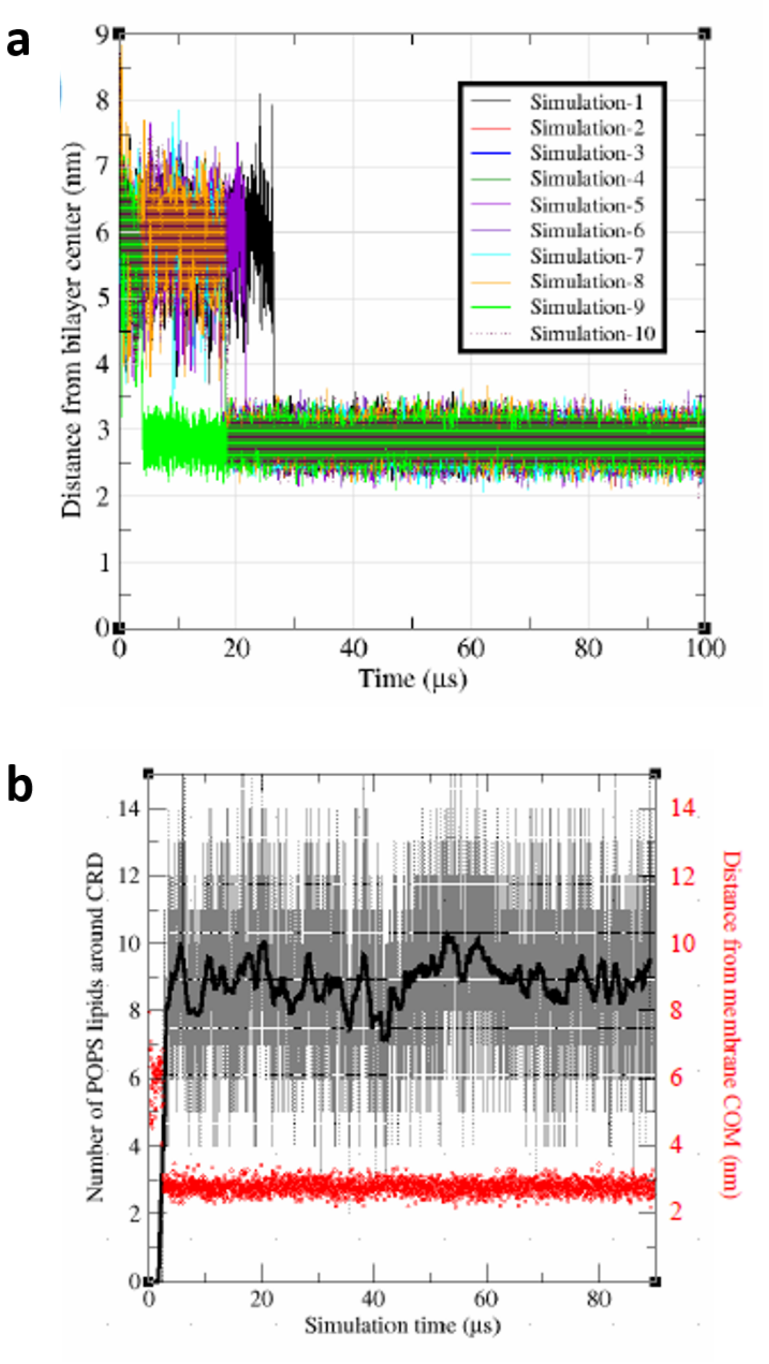

Supp. Figure 16. (a) Time profiles of inter-COM distance between CRD and membrane in the CG simulations of the membrane-anchored docked model S1. (b) Time profiles of POPS lipid counts (black curve) around the CRD in the CG simulations of the membrane-anchored docked model S1.

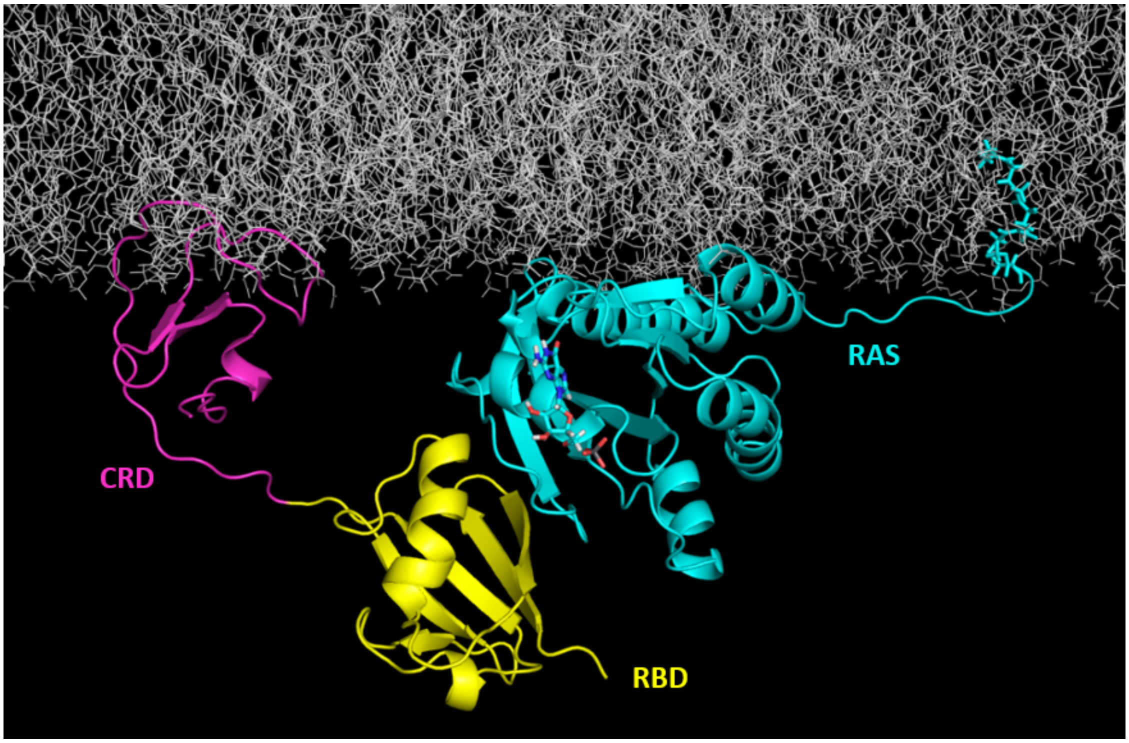

Supp. Figure 17. Starting configuration for RAS/RBD/CRD simulations with CRD initially positioned approximately 2 nm away from RAS/RBD.

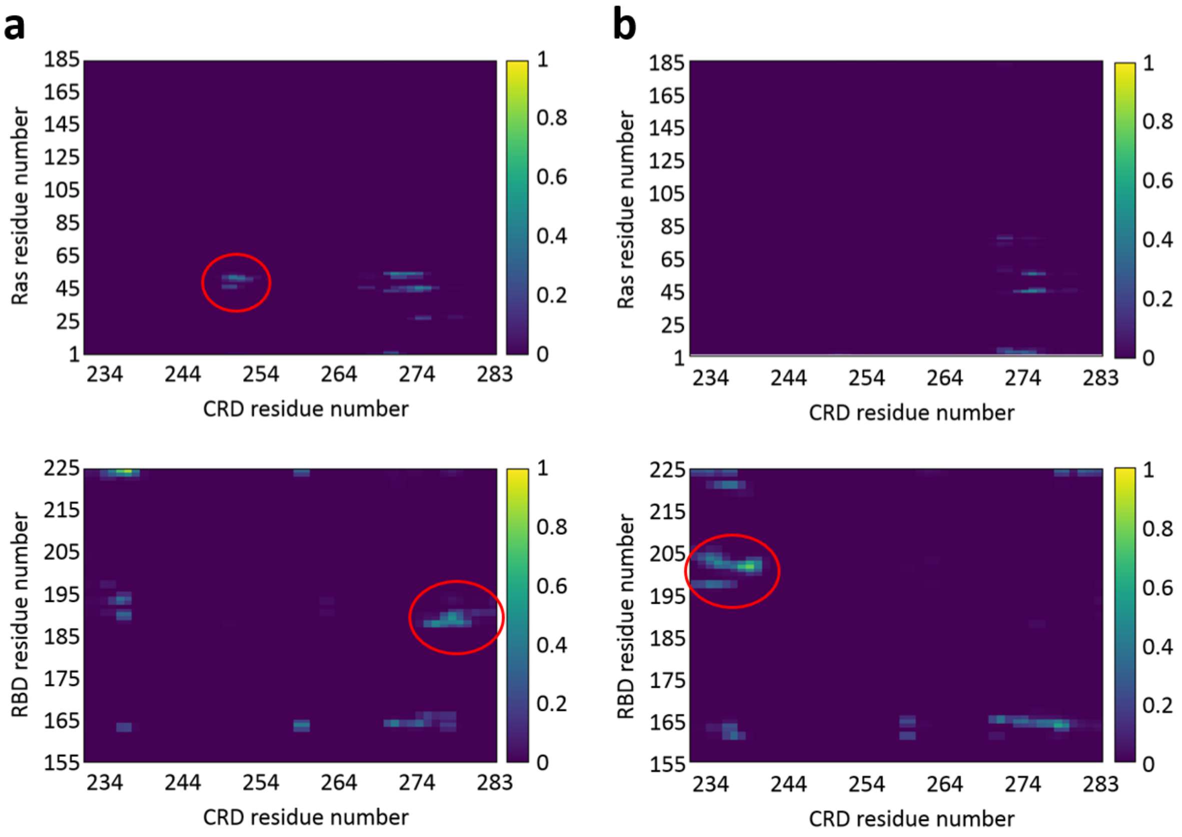

Supp. Figure 18. Residue contact maps for the (a) Membrane-adjacent/GH5 and (b) Membrane-adjacent/GH3 orientations of the ternary complex, between (top) RAS/CRD and (bottom) RBD/CRD, based on CG simulations of membrane-anchored RAS/RBD/CRD with CRD initially positioned 2 nm away from RAS/RBD. Red circles indicate unique contact regions observed between each domain pair.

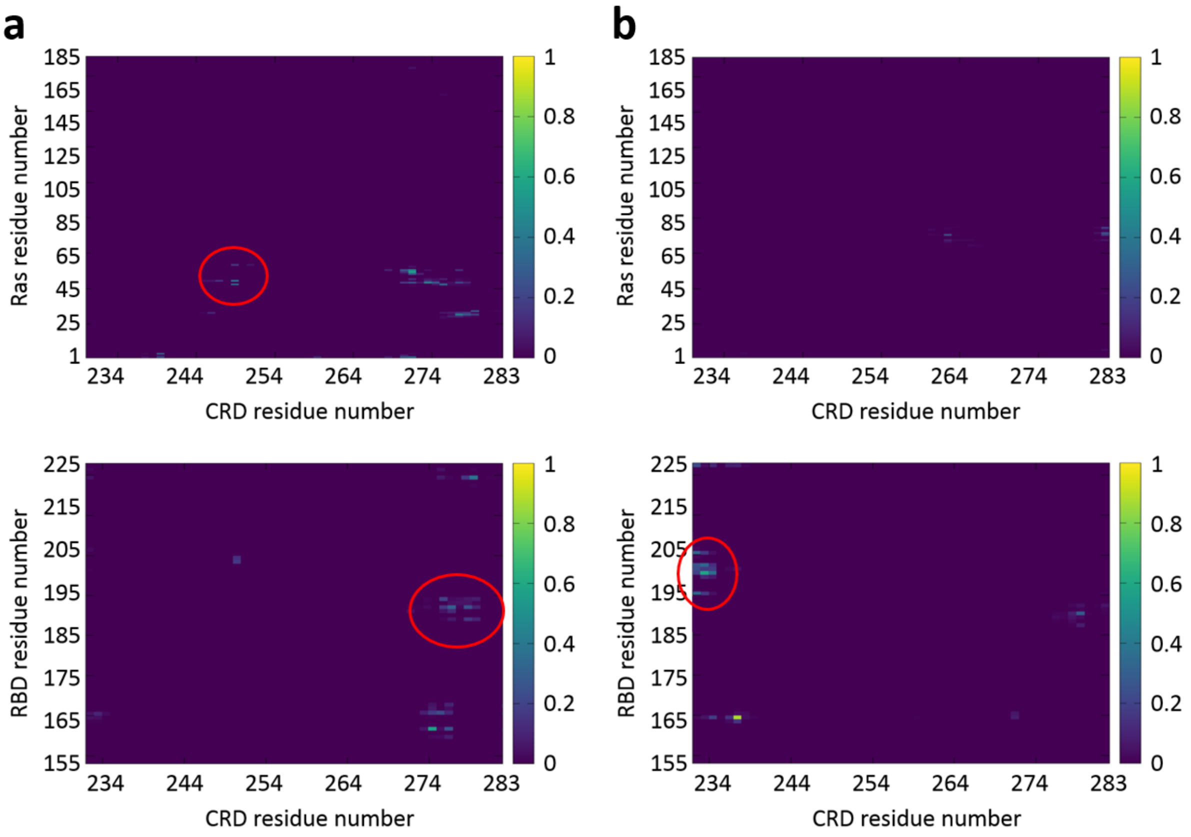

Supp. Figure 19. Residue contact maps for the (a) Membrane-adjacent/GH5 and (b) Membrane-adjacent/GH3 orientations of the ternary complex, between (top) RAS/CRD and (bottom) RBD/CRD, based on AA simulations of backmapped configurations for both orientations from CG simulations of membrane-anchored RAS/RBD/CRD with CRD initially positioned 2 nm away from RAS/RBD. As in **Supp. Figure 18**, red circles indicate unique contact regions observed between each domain pair.

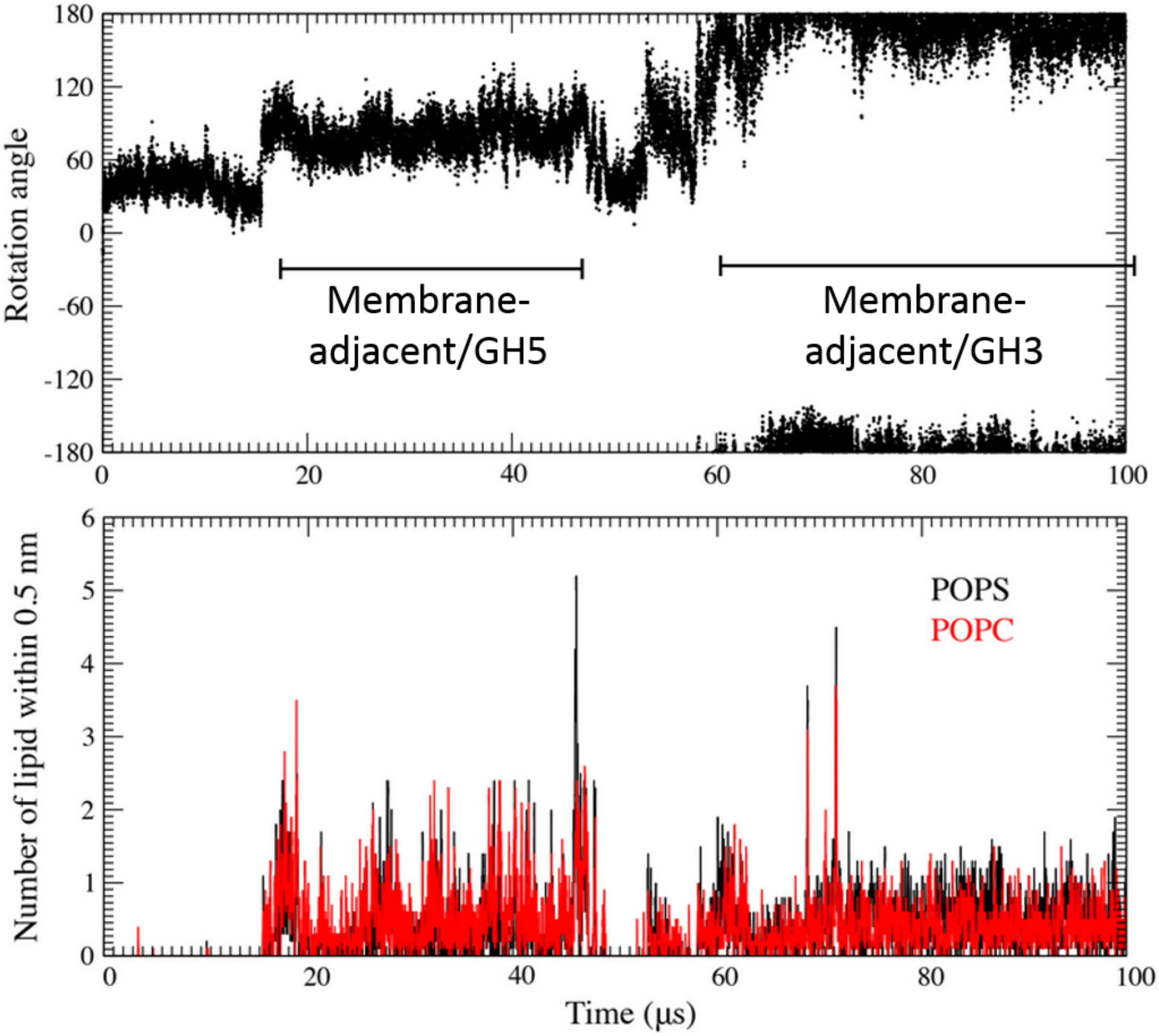

Supp. Figure 20. Time profiles for the rotation angle order parameter (top) and count of POPC/POPS lipid interactions with RBD (bottom) for frames from one of the CG simulations of membrane-anchored RAS/RBD/CRD with CRD initially positioned 2 nm away from RAS/RBD.

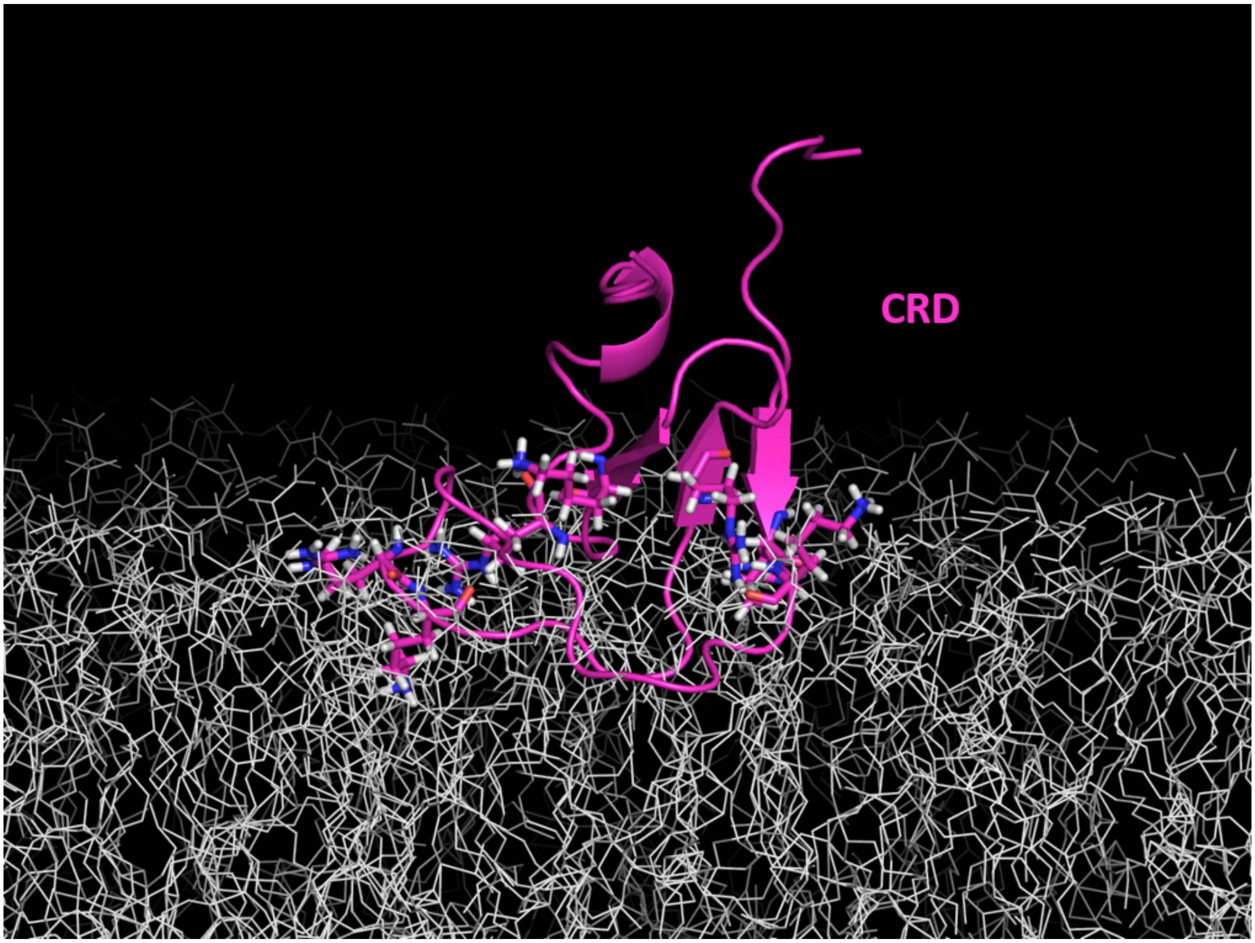

Supp. Figure 21. Snapshot of membrane-anchored CRD showing the “belt” of arginine and lysine residues (magenta sticks) around the two CRD hydrophobic loops and near the lipid head groups.

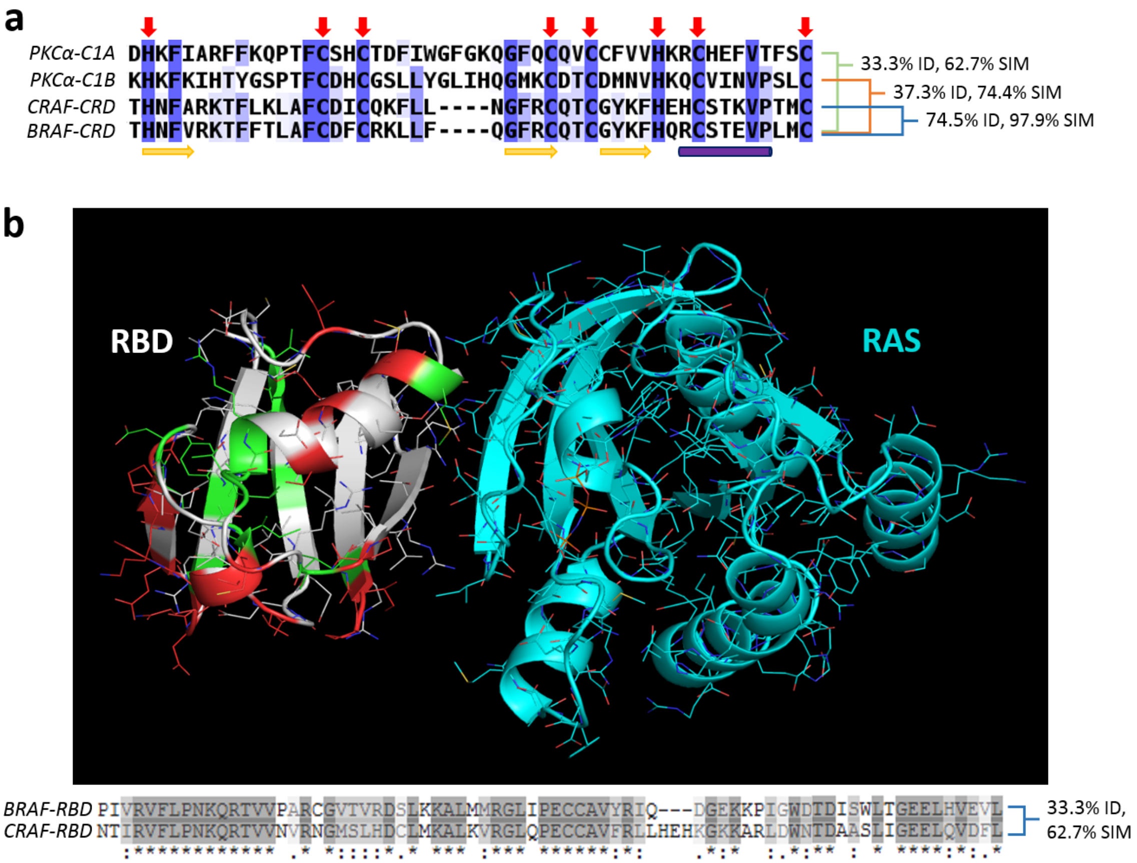

Supp. Figure 22. (a) Multiple sequence alignment between CRD domains of BRAF and CRAF, and homologous C1 domains of protein kinase C alpha (PKCα). Residues belonging to secondary structures are indicated by either by a yellow arrow (β-strand) or a purple cylinder (α-helix). Red arrows indicate conserved cysteine and histidine residues that coordinate either of the two zinc ions. Pairwise sequence identities and similarities with BRAF-CRD are shown on the right. (b) The RAS-binding interface on RBD between BRAF and CRAF is conserved. (top) Structure of the complex between RAS (cyan lines and cartoons) and RBD. Based on comparison between BRAF and CRAF sequences of RBD, the RBD structure is colored here such that conserved residues, chemically similar mutations (such as V to L), and chemically dissimilar mutations (such as R to L) are colored white, green, and red, respectively. (bottom) Multiple sequence alignment between RBD domains of BRAF and CRAF. Pairwise sequence identity and similarity is shown on the right.

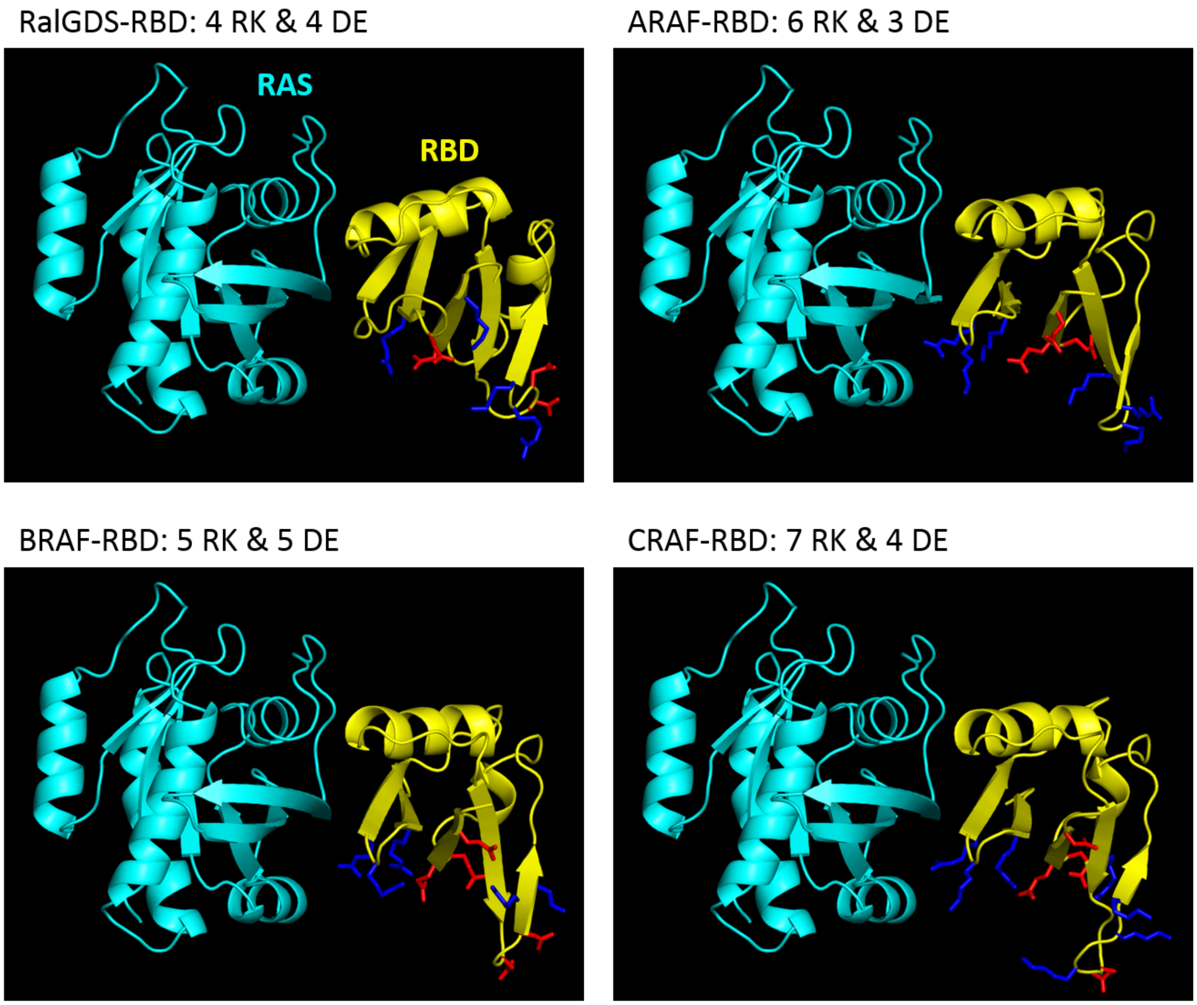

Supp. Figure 23. The membrane-binding interface of RBD (yellow cartoons) in the Membrane-adjacent orientations has a net neutral charge for RalGDS-RBD (top-left) and BRAF-RBD (bottom-left), and a net positive charge for ARAF-RBD (top-right) and CRAF-RBD (top left). Positively-charged and negatively-charged residues on this interface are shown as blue and red sticks, respectively. RK: arginine and lysine residues, DE: aspartic acid and glutamic acid residues.

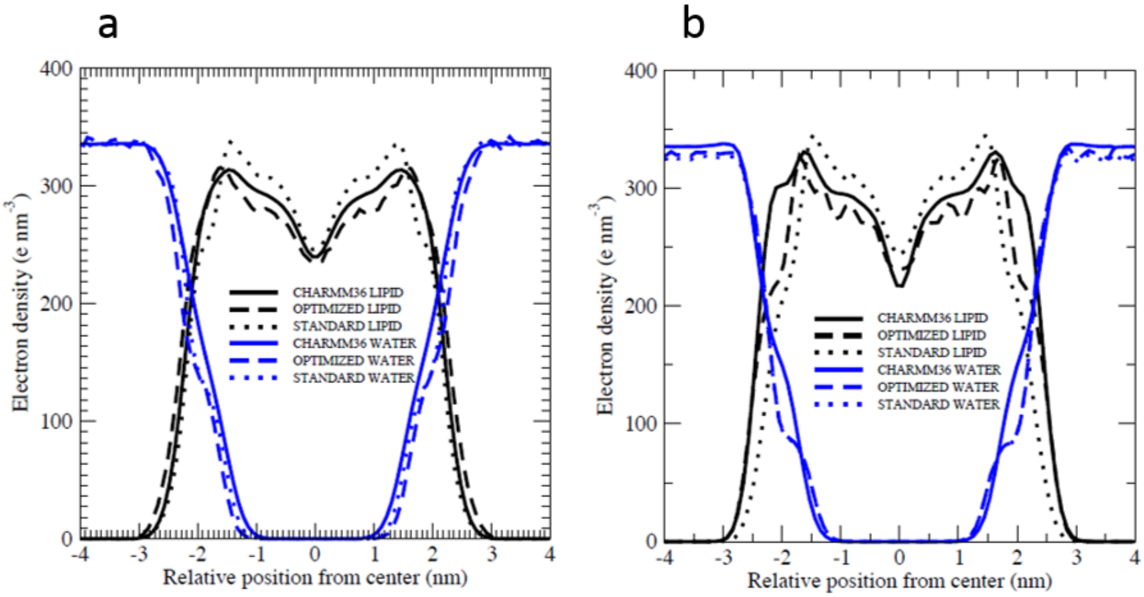

Supp. Fig. 24. Comparison of electron density profiles computed using CHARMM36 (solid lines), standard Martini (dotted lines), and reparametrized/optimized Martini (dashed lines) for membrane simulations of pure POPC (a) or pure POPS (b).

a

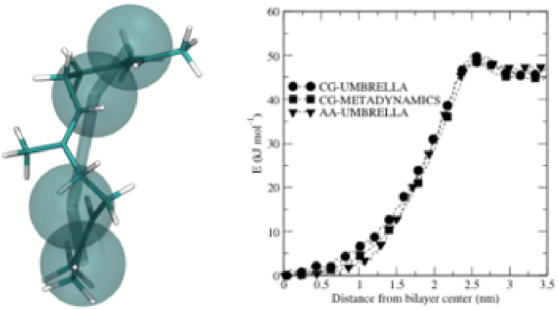

Δoct kJ/mol	Δwat kJ/mol	Log(P)
AA -31	14	7.7
CG -50.78	-0.72	8.6

b

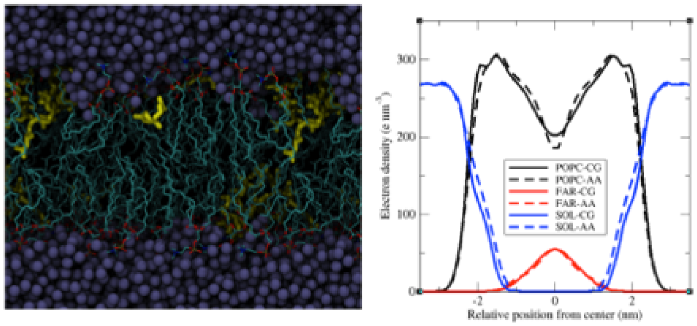

Supp. Fig. 25. (a) Top-left, the CG representation of the farnesyl group (cyan spheres) overlaid on its AA structure. Top-right, PMF of membrane permeation for the farnesyl group computed from AA umbrella sampling (triangles), CG umbrella sampling (circles), and CG parallel tempering metadynamics (squares). Bottom, Computed values of octanol and water solvation free energies and octanol-water partition coefficients from AA and CG simulations. (b) Left, Snapshot from an AA simulation showing the structures and localizations of farnesyl (yellow sticks) in a solvated POPC membrane patch (POPC shown as cyan lines, water shown as blue spheres). Right, electron density profiles for farnesyl (red), POPC (black), and water (blue) from AA (dashed lines) and CG (solid lines) simulations.
